# Reduction of eEF2 kinase alleviates the learning and memory impairment caused by acrylamide

**DOI:** 10.1186/s13578-024-01285-7

**Published:** 2024-08-23

**Authors:** Xiao-Li Wang, Ru-Nan Zhang, Yu-Lin Pan, Zhi-Ming Li, Hong-Qiu Li, Ya-Ting Lei, Fang-Fang Zhao, Xiao-Xiao Hao, Wei-Wei Ma, Cui-Ping Yu, Hong-Wei Yao, Xin-Yu Wang, Jun-Jie Lv, Yong-Hui Wu, Sheng-Yuan Wang

**Affiliations:** 1 Department of Occupational Health, Public Health College, Harbin Medical University, 157 Baojian Road, Nan gang District, 150086 Harbin, People’s Republic of China; 2 Department of Nutrition and Food Hygiene, National Key Discipline, Harbin Medical University, Harbin, People’s Republic of China; 3 Harbin Railway Center for Disease Control and Prevention, Harbin, People’s Republic of China

**Keywords:** Acrylamide, Occupational exposure, eEF2K, Learning and memory, Ether lipid metabolism

## Abstract

**Background:**

The impact of acrylamide (ACR) on learning and memory has garnered considerable attention. However, the targets and mechanisms are still unclear.

**Results:**

Elongation factor 2 (eEF2) was significantly upregulated in the results of serum proteomics. Results from in vitro and in vivo experiments indicated a notable upregulation of Eukaryotic elongation factor 2 kinase (eEF2K), the sole kinase responsible for eEF2 phosphorylation, following exposure to ACR (*P* < 0.05). Subsequent in vitro experiments using eEF2K siRNA and in vivo experiments with eEF2K-knockout mice demonstrated significant improvements in abnormal indicators related to ACR-induced learning and memory deficits (*P* < 0.05). Proteomic analysis of the hippocampus revealed Lpcat1 as a crucial downstream protein regulated by eEF2K. Kyoto Encyclopedia of Genes and Genomes (KEGG) pathway enrichment analyses indicated that eEF2K may play a role in the process of ACR-induced learning and memory impairment by affecting ether lipid metabolism.

**Conclusions:**

In summary, eEF2K as a pivotal treatment target in the mechanisms underlying ACR-induced learning and memory impairment, and studies have shown that it provides robust evidence for potential clinical interventions targeting ACR-induced impairments.

**Supplementary Information:**

The online version contains supplementary material available at 10.1186/s13578-024-01285-7.

## Introduction

Acrylamide (ACR) is a low-molecular-weight, highly hydrophilic vinyl monomer. ACR is widely present in coffee, potato chips, biscuits, and high-temperature foods prepared under low-humidity conditions [1, 2]. Additionally, ACR and its polymers are extensively used in industrial production, including the manufacturing of dyes, organic chemicals, paper, and textiles, etc. [3–5]. ACR can cause neurotoxicity, genotoxicity, carcinogenic toxicity, hepatotoxicity and reproductive toxicity in animals [6–9]. However, only ACR neurotoxicity has been found in humans [10, 11]. The World Health Organization report shows that the impact of ACR on the nervous system of the population in current occupations and environments has become a practical issue of concern to the occupational health field and the government [12].

Studies suggest that ACR may cause harm to both the peripheral and central nervous systems [13–15]. The current research on peripheral nervous system damage caused by ACR has become relatively mature. Mechanisms underlying ACR-induced peripheral nervous system damage include oxidative stress, inflammatory responses, DNA damage, and neurotransmitter imbalances [16–18]. In both in vitro studies and cases of occupational poisoning, it has been found that ACR can lead to peripheral nervous system symptoms such as numbness in the hands and feet, muscle weakness, loss of vibratory sensation, and ataxic gait [19, 20]. However, there is a growing interest among researchers on the impact of ACR in the central nervous system, particularly its role in affecting learning and memory functions.

In a prospective cohort epidemiological study of 2534 non-smoking older adults, those exposed to ACR had a significantly increased risk of memory deficits and cognitive dysfunction, and males were more susceptible [21]. In vivo studies have shown that repeated exposure to ACR could lead to degeneration in brain areas associated with learning, memory and cognitive function [22]. Similarly, studies have shown that exposure to ACR for 16 weeks exacerbates cognitive deficits in mice, while chronic exposure to ACR leads to neuronal impairments in the hippocampus and cortex, resulting in poor performance in spatial memory tests [23].

However, there has been limited research on the targets that ACR plays an important role in causing learning and memory impairment. Therefore, the mechanisms of ACR in causing learning and memory impairment remain unclear. Although some studies have examined the mechanisms of ACR in causing learning and memory impairment, they were conducted in vitro and animal experiments, and there have been very few population-based studies [24, 25]. In our previous research, our group established a stable occupational cohort exposed to ACR and conducted a series of studies in this population [26–29]. Therefore, we will study the possible underlying mechanisms of ACR in the process of causing learning and memory impairment based on a stable ACR occupational population.

Firstly, we used proteomics technology to analyze the serum of ACR occupationally exposed groups and screen out the target proteins that ACR may affect in causing learning and memory impairment. Then we conducted multi-dimensional experimental research in vivo and in vitro to verify the role of the selected target proteins in ACR-induced learning and memory impairment, and analyzed the main molecular mechanisms of the impact. Identifying a new important target for learning and memory impairment caused by ACR will provide an important theoretical basis for the prevention or treatment of central nervous system damage caused by ACR.

## Materials and methods

### Chemicals, reagents and antibodies

Amresco (USA) supplied ACR with a purity of 99.9%. DMEM medium was purchased from Procell (China). Fetal bovine serum (FBS) was purchased from Wenren. 4% paraformaldehyde was purchased from Biosharp (China). Glutaraldehyde fixative solution (2.5%, for electron microscopy) and 50×TAE were purchased from LEAGENE (China). DAPI dye, BCA protein assay kit, and cell counting kit-8 (CCK-8) were obtained from Beyotime (China). eEF2K-siRNA was sourced from RIBOBIO (China). The PAGE Gel Fast Preparation kit and RT-qPCR assay kit were purchased from Epizyme (China) and Toyobo (Japan), respectively. The gene identification kit was purchased from TIANGEN (China).

The primary antibodies included eukaryotic elongation factor 2 (eEF2) (Cat. ABP58457, Abbkine), Phospho-eEF2 (Thr56) antibody (Cat. ABP58456, Abbkine), eEF2K antibody (Cat. GTX111496, GeneTex), Synapsin 1 (SYN1) (Cat. ABP0125, Abbkine), tropomyosin receptor kinase B (TrkB) (Cat. ABP55433, Abbkine), brain-derived neurotrophic factor (BDNF) (Cat. GTX132621, GeneTex), lysophosphatidylcholine acyltransferase 1 (Lpcat1) (Cat. DF12033, Affinity), β-actin (Cat. TA811000, ORIGENE). The secondary antibodies used were FITC-conjugated goat anti-mouse IgG (Cat. TA130013, ORIGENE) and Cy3-conjugated goat anti-rabbit IgG (Cat. A23920, Abbkine). HRP-conjugated antibodies included goat anti-mouse IgG (Cat. A21010, Abbkine) and goat anti-rabbit IgG (Cat. A21020, Abbkine).

### Study subjects

We defined the workers in the ACR monomer production workshop of a factory in northern China as the ACR exposure group for this study, and the administrative workers in the same factory, who were strictly not exposed to ACR, as the ACR non-exposed group. We investigated the basic situation of the factory, hygiene and protective measures, as well as personal protective equipment. A standardized questionnaire was used to record the general information, occupational history, and personal protective measures of the survey subjects. We also investigated the occurrence of subjective symptoms related to learning and memory in the ACR-exposed group and the non-exposed group. The questionnaire survey was conducted face-to-face to ensure independent and careful completion by each respondent, minimizing survey result bias. Exclusion criteria for the study encompassed long-term use of antipsychotic drugs, systemic diseases impacting the central nervous system’s function, metabolic disorders, other conditions affecting blood metabolites, and diabetes resulting from prolonged medication use. Approval for the study was granted by the Ethical Committee of Harbin Medical University, and all subjects provided informed consent [26–29].

A total of 100 people were surveyed in the study, including the ACR-exposed group (*n* = 50; mean age, 42.16 ± 4.39 years; mean working years, 16.62 ± 5.24 years) and the non-exposed group (*n* = 50; mean age, 41.64 ± 3.47 years; mean working years, 16.20 ± 2.46 years). We randomly selected 10 individuals with subjective symptoms related to learning and memory from the ACR-exposed group (*n* = 50), and 10 individuals without self-reported symptoms related to learning and memory from the non-exposed group (*n* = 50). The selected ACR-exposed group (*n* = 10; age, 42.10 ± 3.98 years; working years, 17.02 ± 4.37 years) and non-exposed group (*n* = 10; age, 43.80 ± 3.85 years; working years, 18.00 ± 2.11 years) were subjected to serum proteomic analysis.

### Serum collection

Both groups adhered to identical dietary restrictions and refrained from alcohol consumption for the three days preceding the collection of blood samples. Morning fasting cubital venous blood (4 mL) was collected from all subjects using blood collection tubes with sodium heparin. The blood underwent rapid centrifugation at 3000×g for 10 min at room temperature, leading to the separation of serum, which was then stored at -80 °C until further analysis.

### Population serum proteomics

The serum samples were taken out from − 80 °C and centrifuged at 4 °C, 12,000×g for 10 min. 600 µg of the supernatant was taken and the Pierce™ Top 12 Abundant Protein Depletion Spin Columns Kit from Thermo was used according to the instructions to remove high-abundance proteins. The protein concentration was determined using the BCA assay kit. The proteins were then enzymatically digested into peptides using trypsin, followed by desalting with Strata X C18 (Phenomenex) and vacuum freeze-drying. The peptides were dissolved in 0.5 M TEAB and labeled according to the instructions of the TMT assay kit. The labeled peptides were analyzed using liquid chromatography mass spectrometry, and the results were finally organized and analyzed (Supplementary Method 1).

### Cell culture and transfection

Well-differentiated rat pheochromoma cells (PC12) were obtained from Procell Life Science & Technology Co., Ltd., Wuhan, China. The cells were cultured in cell culture medium containing 10% fetal bovine serum, 1% penicillin/streptomycin, and 89% DMEM. The cells were maintained at 37℃ in a 5% CO_2_ incubator and passaged using Trypsin digestion solution for sub-culturing.

PC12 cells were mainly used for two parts of research. The first part of the research is to expose PC12 cells to different concentrations of ACR solutions to observe the expression of eEF2K in PC12 cells. PC12 cells were divided into control group, 1.25 mM ACR group and 2.5 mM ACR group, and the exposure time was 24 h. The second part of the research is to use eEF2K-siRNA to inhibit the expression of eEF2K in PC12 cells and explore whether inhibiting the expression of eEF2K in PC12 cells can improve the neurotoxicity caused by ACR. The PC12 cells were divided into four groups: the control group, the eEF2K-siRNA group (final concentration 10 µM), the ACR group (2.5 mM ACR), and the combined eEF2K-siRNA (final concentration 10 µM) and ACR (2.5 mM ACR) group. The exposure duration for each group was 24 h. Lipofectamine 2000 (Invitrogen, Carlsbad, CA, USA) was employed for cell transfection. siRNA used in the transfection were synthesized by Thermo Fisher Scientific (Massachusetts, USA).

### Animal feeding and grouping

Eighteen 8-week-old SPF male SD rats were purchased from Vital River Laboratories (Beijing, China). The license number is SCXK (JING) 2021-0011. eEF2K heterozygous C57BL/6J mice (eEF2K^+/−^) were purchased from Gempharmatech Co., Ltd (Jiangsu, China), and the strain number is T037867. We obtained wild-type (WT) and KO (eEF2K^−/−^) pups by crossing heterozygous mice (eEF2K^+/−^). Twenty-four 8-week-old male WT mice and Twenty-four 8-week-old male KO mice were used for experimental studies. All rats and mice were subjected to 1 week of acclimatization feeding and were housed under a 12:12 h light/dark cycle, at a temperature of 22 ± 1 °C and a relative humidity of 55 ± 10%. They had ad libitum access to food and water. All procedures adhered to the regulations set by the Ethical Committee for Research on Laboratory Animals, as reviewed and approved by the Medical Ethics Committee of Harbin Medical University (Harbin, China). The approval process aligns with guidelines established by the National Institutes of Health (USA) [27].

Eighteen male SD rats were randomly assigned to three groups: control, low-dose ACR, and high-dose ACR (*n* = 6 per group). The low-dose ACR group received a dose of 6 mg/kg body weight, while the high-dose ACR group received a dose of 18 mg/kg body weight. The control group was administered distilled water. Twenty-four male WT mice and 24 male KO (eEF2K^−/−^) mice were randomly divided into four groups, including WT group, KO group, WT + ACR group, and KO + ACR group (*n* = 12 each group). The WT group and KO group were given distilled water, while the WT + ACR group and KO + ACR group were given a dose of 18 mg/kg body weight of ACR. Both rats and mice were orally gavaged with a dose equivalent to 1% of their body weight, and the duration of treatment was 28 days. Following the last oral gavage of ACR, both rats and mice were euthanized, and the entire hippocampal tissue was isolated based on specific experimental needs. The dosage of ACR administered to rats and mice in this study was determined based on our previous research [27].

### Cell viability assay

PC12 cells were evenly distributed in a 96-well plate, with each well containing 1 × 10^4^ cells/mL. The experimental group and eEF2K transfection group were treated separately. Each concentration group was set up with 6 duplicate wells and cultured at 37 °C and 5% CO_2_ for 24 h. 10 µL of CCK-8 solution was added to each well 1 h before the end, and the culture was continued in the incubator for 2 h. The absorbance was measured using a microplate reader at 490 nm wavelength. The cell survival rate is presented as the relative survival rate, calculated as the ratio of the absorbance value in the experimental group and eEF2K transfection group to the absorbance value in their corresponding control group.

### Behavior tests

Rats and mice were subjected to behavioral tests continuously within 3 days after completing the exposure. *Open field test.* The open field test equipment included an open field box and a top camera. The equipment was purchased from Shenzhen Ward Life Science Co., Ltd., China. The activity of each rat was individually monitored for 10 min in the open field (L × W × H: 100 × 100 × 40 cm). The activity of each mouse was individually monitored for 5 min in an open field (L × W × H: 50 × 50 × 40 cm*).* All data were recorded using video-tracking software (SMART, Panlab Harvard Apparatus Bioscience Company, USA). Parameters evaluated included Movement time in Periphery and Resting Time in Zone (s) - Center. *Shuttle Box Test.* The experiment used a shuttle box with dimensions of 696 × 348 × 445 mm (Chengdu Taimeng Technology Co., Ltd., China). Mice were given 2 min to adapt in the shuttle box, including walking freely in the shuttle box to become familiar with the learning environment. At the experiment’s outset, position the mouse in the left compartment of the shuttle box, near and facing the end wall. If the mouse relocates to the opposite compartment during the conditioned stimulus (CS), the conditioned response is automatically logged. The CS lasted for a maximum of 10 s, followed by an unconditioned stimulus (US) for a maximum of 10 s. Mice were studied for three consecutive days and tested on the fourth day. STT-100 shuttle experiment video analysis software was used to analyze and make statistics on the experimental data. The parameters evaluated include the percentage of conditioned responses and the percentage of unconditioned responses.

### Histopathological observation of hippocampus

Rats and mice were anesthetized and subjected to transcardial perfusion with 200 mL of cold normal saline (4 °C) followed by 300 mL of ice-cold 4% paraformaldehyde (PFA) (4 °C). Rats and mice hippocampal tissues were fixed in 4% PFA, dehydrated, and embedded in paraffin. Hippocampal tissues were sectioned into 4 μm-thick serial sections and stained with hematoxylin and eosin (HE). Each slide received 50 µL of neutral gum, and the sections were examined under a light microscope (BH-2; Olympus Corporation, Tokyo, Japan). A pathologist, blinded to group assignment, conducted the histological examination.

### Immunohistochemical analysis

The fixation methods of rat and mouse hippocampal tissues were the same as those for histopathological observation. Following the fixation of hippocampal tissues, the tissues were immersed in 30% sucrose in 0.01 M PBS until they sank to the bottom of the container. Brains were then mounted on poly-L-lysine-coated slides and sliced into 4 µM sections using a cryostat. These sections underwent a 30-minute incubation with 2% normal goat serum to prevent nonspecific binding. Then the diluted primary antibody was added dropwise, and rat hippocampal tissues were incubated with eEF2K (1:500) and mouse hippocampal tissues were incubated with eEF2K (1:300) overnight (15 h) at 4 °C. After washing three times with PBS, HRP-labeled goat anti-rabbit/mouse secondary antibody was added dropwise and incubated at 37 °C for 30 min. The sections were detected using a DAB color development kit (Servicebio, China). 50 µL of neutral gum was added to each slide, and the slides were observed under a microscope (Fi3, Nikon, Japan). Subsequently, image analysis was performed.

### Immunofluorescence analysis

The fixation and sectioning procedures for rat hippocampal tissues mirrored those employed for histopathological observation and immunohistochemical analysis. Following completion, the sections underwent antigen retrieval (4.75 mL antigen retrieval agent and 500 mL double-distilled water), were boiled for 10 min, and then washed three times with PBS for 5 min each. Subsequently, 100 µL of blocking solution was added, and the sections were incubated at room temperature for 30 min. We added rabbit anti-rat primary antibody eEF2K (1:500) to the samples and incubated them overnight at 4 °C. After three PBS washes, slices were treated with a dark incubation of fluorescent (Cy3)-labeled goat anti-rabbit IgG secondary antibody for 60 min. Subsequently, three additional PBS washes, each lasting 5 min, were performed. DAPI was introduced and allowed to incubate for 5 min in the dark, followed by three PBS washes, each lasting 5 min. Images were observed and captured using a fluorescence microscope (Fi3, Nikon, Japan).

PC12 single cell suspension was prepared, and the cell concentration was adjusted to 1 × 10³ cells/mL before being inoculated in a 6-well plate. After the cells were adherent and grown, they were divided into experimental groups and treated with poison for 24 h. 4% PFA was added for fixation for 30 min and then 0.1% Triton-100 was added for permeabilization for 30 min. After a 30 min blocking step with 2% BSA, rabbit anti-rat eEF2K (1:1000) was applied and incubated overnight at 4 °C. The secondary antibody (goat anti-rat IgG-FITC) was introduced, followed by a 1 h incubation at 37 °C, and DAPI was added for 10 min. Each step involved three washes with PBS. Subsequently, images were observed and captured for analysis using a fluorescence microscope (Fi3, Nikon, Japan).

### Observation of morphology in hippocampal tissue and PC12 cells using transmission Electron Microscopy

Rat and mouse hippocampal tissues, along with PC12 cells, were transformed into sample blocks measuring 1 mm³ and promptly immersed in 2% glutaraldehyde fixative for 2 h. The tissues underwent three washes with 0.1 M phosphate buffer (pH = 7.4), each lasting 15 min. Following a 1 h treatment with 1% osmic acid, the samples were rinsed three times with 0.1 M phosphoric acid solution, with each rinse lasting 15 min. Ethanol and acetone were employed for a 15 min gradient dehydration process. The tissue was embedded in a porous rubber embedding template (embedding agent: anhydrous ethanol = 1:1) and subsequently dried in an oven to create an embedding block. Sections, approximately 70 nm in thickness, were then produced using an ultramicrotome (Leica Ultracet R, Germany). Ultimately, double staining with 3% uranyl acetate and lead citrate was performed, and transmission electron microscopy (JEM1230, Japan) was utilized for image observation and collection for subsequent analysis.

### Western blot analysis

The hippocampal tissues from rats and mice, along with PC12 cells, were lysed using RIPA lysis buffer (Beyotime Biotechnology, Shanghai, China) containing 1% PMSF based on the experimental groups. The sample suspension underwent centrifugation at 4 °C, 12,000×g for 10 min, and the resulting supernatant was collected. Total protein concentration was determined using the BCA protein assay kit (Beyotime Biotechnology, Shanghai, China). Equal aliquots of samples containing 50 g of protein were subjected to 15% (w/v) SDS-polyacrylamide gel electrophoresis and transferred onto polyvinylidene difluoride (PVDF) membranes. The membranes were blocked with 5% skim milk in Tris-buffered saline and Tween 20 (TBST) for 2 h. Following this, blots were incubated overnight at 4 °C with rabbit polyclonal primary antibodies against eEF2K (1:1000), eEF2 (1:1000), p-eEF2 (1:1000), BDNF (1:1000), SYN1 (1:1000), TrkB (1:1000), Lpcat1 (1:500), and β-actin (1:1000). The membranes underwent five washes with TBST and were then incubated at room temperature for 2 h with a secondary antibody (goat anti-mouse/rabbit) diluted at 1:10000. Target proteins were detected using an enhanced chemiluminescence detection kit, and the relative intensity bands were analyzed using a gel imaging analysis system. β-actin served as an internal control.

### RNA extraction and real-time qPCR analysis

RNA extraction from hippocampal tissue (rats and mice) and cell lysates utilized the E.Z.N.A.^®^ Total RNA Kit I (Omega, China), following the manufacturer’s instructions. Subsequent to extraction, RNA underwent reverse transcription using the ReverTra Ace^®^ qPCR RT Master Mix with gDNA Remover (TOYOBO, Japan). The resulting cDNA served as a template for amplification with THUNDERBIRDTM Next SYBR^®^ qPCR Mix (TOYOBO, Japan). Gene expression levels were quantified by normalizing to β-actin. Detailed primer sets for genes are provided in Supplementary Table 1 and Supplementary Table 2.

### Mouse hippocampal tissue proteomics

The appropriate amount of mouse hippocampal tissue was taken out from a -80 °C freezer and was ground. After tissue grinding, the lysate was subjected to centrifugation at 12,000×g for 10 min at 4 °C, and the resulting supernatant was collected for protein concentration determination. Peptide fragments obtained from trypsin digestion were desalted using Strata X C18 (Phenomenex) and subsequently vacuum freeze-dried. The peptides were dissolved in 0.5 M TEAB and labeled following the TMT reagent kit instructions. After dissolving the peptide fragments in mobile phase A, separation was achieved using an EASY-nLC 1200 ultra-high-performance liquid chromatography system. Ionization of the separated peptides was performed in the NSI ion source, and analysis was carried out using an Orbitrap Exploris™ 480 mass spectrometer (ThermoFisher Scientific). Finally, the results were organized and analyzed (Supplementary Method 1).

### Molecular docking and bioinformatics analysis

The HDOCK online platform (http://hdock.phys.hust.edu.cn/) was utilized as the molecular docking program in this study (Supplementary Method 2). We utilized Gene Ontology (GO) terms to analyze differentially expressed proteins identified through proteomic analysis, including Cellular Component (CC), Molecular Function (MF), and Biological Process (BP). For the functional enrichment analysis of GO and pathways, a two-tailed Fisher’s exact test was applied to evaluate the enrichment of differentially expressed proteins among all annotated proteins. Pathway analysis utilized the Kyoto Encyclopedia of Genes and Genomes (KEGG) database (https://www.genome.jp/kegg/). The outcomes of GO enrichment and KEGG analysis were presented using R Studio software. To identify protein-protein interaction networks, the differentially expressed proteins’ database identifiers or protein sequences from the comparative group were cross-referenced with the STRING (v.11.5) database. The protein-protein interaction relationships for differentially expressed proteins were extracted based on a confidence score > 0.4 (middle confidence), and the network was visualized using the Cytoscape tool.

### Statistical analysis

Epidata 3.0 software (http://www.epidata.dk) was employed for data entry and analysis. Results are presented as means ± SD. GraphPad Prism9 software was utilized for the analysis of diverse experimental data, with all experiments independently conducted at least three times. Continuous variable data underwent analysis through independent samples *t*-test or one-way analysis of variance, while categorical variables were assessed using the chi-square test. A *P*-value of ≤ 0.05 was deemed statistically significant.

## Results

### Subject characteristics

In this study, we surveyed a total of 100 people. No statistically significant differences were observed between the ACR exposure group and the non-exposed group in terms of age, working years, smoking, alcohol consumption, coffee consumption, and fried food intake (*P* > 0.05) (Table 1). Individuals in the ACR exposure group utilized personal protective equipment during work, such as disposable latex gloves and dust masks. Detailed ACR concentration measurements in the ACR production workshop can be found in our prior research [29].

**Table 1:** Demographic data of the study subjects

Parameter	Exposed group (*n* = 50)	Non-exposed group (*n* = 50)	*P*
Personal characteristics ^a^ mean ± SD			
**Age (years)**	42.16 ± 4.39	41.64 ± 3.47	0.51
**Work years (years)**	16.62 ± 5.24	16.2 ± 2.46	0.61
**Use of personal protective equipment**,** n (%)**	50(100.00)	-	-
**Male** ^**b**^ **(n%)**	39(78.00)	42(84.00)	0.44
**Habitual status**,** n (%)**			
**Coffee consumption** ^**b**^	29(58.00)	34(68.00)	0.30
**Preference for fried food** ^**b**^	47(94.00)	49(98.00)	0.31
**Smoking** ^**b**^	16(32.00)	25(50.00)	0.067
**Alcohol consumption** ^**b**^	33(66.00)	34(68.00)	0.30

^a^ Parameter values of exposed group and non-exposed group were compared using *t*-test

^b^ Parameter values of exposed group and non-exposed group were compared using χ^2^ test

According to the literature review, the subjective symptoms related to learning and memory in this survey mainly include memory loss, slow reaction, dizziness, headache, dreaminess, hearing loss, vision loss and depression [30, 31]. The findings indicated a higher occurrence of subjective symptoms related to learning and memory in the ACR exposure group compared to the non-exposed group (*P* < 0.05) (Table 2). Among the subjects who had serum proteomics, there were no statistically significant differences between the ACR-exposed group and the non-exposed group in terms of age, working years, smoking, alcohol consumption, drinking coffee and eating fried foods (*P* > 0.05) (Table 3).

**Table 2:** Conscious symptoms of learning and memory dysfunction in acrylamide-occupied workers

Parameter	Exposed group (*n* = 50)	Non-exposed group (*n* = 50)	*P*
Subjective symptoms (*n*%)			
**Hypomnesia** ^**b**^	39(78.00)	27(54.00)	0.01
**Unresponsive** ^**b**^	25(50.00)	5(10.00)	< 0.0001
**Giddy** ^**b**^	31(62.00)	14(28.00)	< 0.001
**Headache** ^**b**^	27(54.00)	5(10.00)	< 0.0001
**Dreaminess** ^**b**^	28(56.00)	7(14.00)	< 0.0001
**Hearing loss** ^**b**^	32(64.00)	6(12.00)	< 0.0001
**Decreased vision** ^**b**^	28(56.00)	8(16.00)	< 0.0001
**Melancholy** ^**b**^	16(32.00)	7(14.00)	0.03

^b^ Parameter values of exposed group and non-exposed group were compared using χ^2^ test

**Table 3:** Demographic data of 20 study subjects participating in serum proteomics

Parameter	Exposed group (*n* = 10)	Non-exposed group (*n* = 10)	*P*
Personal characteristics ^a^ mean ± SD			
**Age (years)**	42.10 ± 3.98	43.80 ± 3.85	0.34
**Work years (years)**	17.20 ± 4.37	18.00 ± 2.11	0.61
**Use of personal protective equipment**,** n (%)**	10(100.00)	-	-
**Male** ^**b**^ **(n%)**	5(50.00)	5(50.00)	1.00
**Habitual status**,** n (%)**			
**Coffee consumption** ^**b**^	9(90.00)	10(100.00)	0.18
**Preference for fried food** ^**b**^	10(100.00)	9(90.00)	0.30
**Smoking** ^**b**^	2(20.00)	2(20.00)	1
**Alcohol consumption** ^**b**^	2(20.00)	5(50.00)	0.16

^a^ Parameter values of exposed group and non-exposed group were compared using t-test

^b^ Parameter values of exposed group and non-exposed group were compared using χ^2^ test

### Population serum proteomics results and eEF2K expression in PC12 cells after ACR exposure

**Fig. 1:**
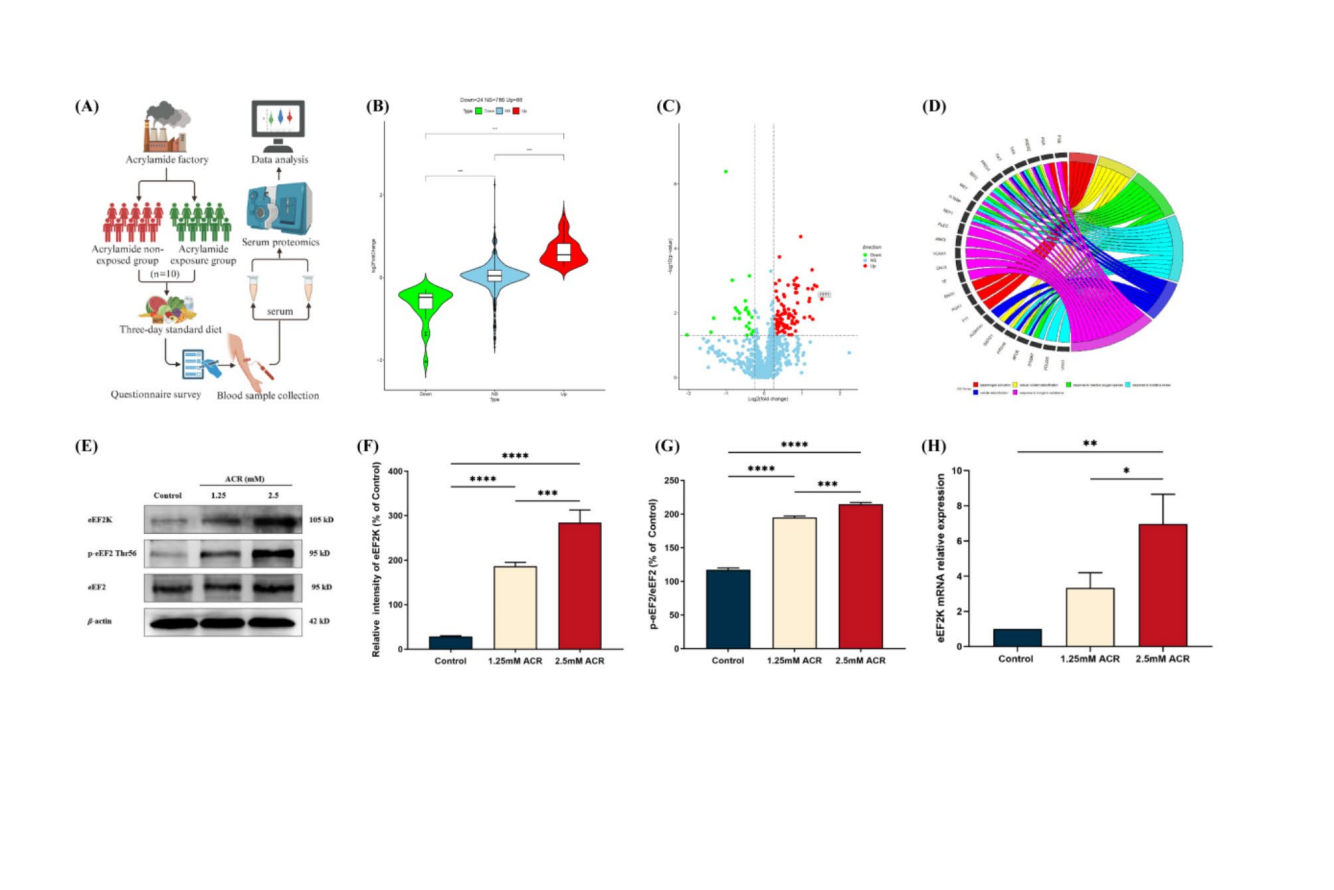
Serum proteomic analysis of ACR occupational population and expression of eEF2 and eEF2K in PC12 cells. **(A)** Flow chart of experimental design of serum proteomics in ACR-exposed and non-exposed groups. **(B)** Differential protein expression in serum proteomics between ACR-exposed and non-exposed groups. Fold change ≥ 1.2, ^***^P < 0.001. **(C)** Volcano plot of differentially expressed proteins between ACR-exposed and non-exposed groups. Among them, eEF2 expression was significantly upregulated. **(D)** Chord plot of GO enrichment analysis of differentially expressed proteins between the ACR exposed group and the non-exposed group. **(E)** Effects of ACR on the expression of eEF2, eEF2K, and p-eEF2 proteins in PC12 cells. **(F)** The protein expression levels of eEF2K in ACR-infected PC12 cells. Data are expressed as means ± SD, *n* = 3. ^***^P < 0.001, ^****^P < 0.0001. **(G)** The protein expression levels of p-eEF2/eEF2 in ACR-infected PC12 cells. Data are expressed as means ± SD, *n* = 3. ^***^P < 0.001, ^****^P < 0.0001. **(H)**The expression levels of eEF2K mRNA after ACR-infected PC12 cells. Data are expressed as means ± SD, *n* = 3. ^*^P < 0.05, ^**^P < 0.01

We performed serum proteomics analysis in ACR-exposed and non-exposed groups (Fig. 1A). Principal component analysis (PCA) results showed that the ACR-exposed and non-exposed groups were significantly separated (Supplementary Fig. 1A). The proteomics research results were screened and analyzed for differential proteins according to the principles of Fold change ≥ 1.2 and *P* < 0.05. Compared with the ACR non-exposed group, there were 88 upregulated differential proteins, 24 downregulated differential proteins, and 786 no differential proteins in the ACR-exposed group (Fig. 1B). We performed GO enrichment and KEGG analysis on 112 upregulated and downregulated differential proteins. The results of GO enrichment indicate that ACR may affect the function of phospholipid efflux (Supplementary Fig. 1B). The results of the KEGG enrichment suggested that ACR may be involved in cholesterol metabolism (Supplementary Fig. 1C). The results of the volcano plot and differential protein interaction network diagram showed that there were 88 upregulated differential proteins in the ACR-exposed group, among which eEF2 was the most significantly upregulated and had a strong interaction with other differential proteins. We used a chord diagram to display the functional enrichment of eEF2, and the results indicated that the main functional enrichments of eEF2 were in response to reactive oxygen species, oxidative stress and inorganic substance (Fig. 1D).

Due to eEF2K being the only kinase responsible for phosphorylating eEF2, we conducted in vitro experiments to verify whether exposure to ACR affects the protein expression of eEF2, eEF2K, and p-eEF2 in PC12 cells. Compared with the control group, the higher the ACR concentration was, the higher the expressions of eEF2K and p-eEF2/eEF2 were (*P* < 0.05) (Fig. 1E, F and G). RT-qPCR results further demonstrated a dose-dependent elevation in eEF2K mRNA expression with increasing ACR exposure (*P* < 0.05) (Fig. 1H).

### Effects of ACR exposure on hippocampal tissue dentate gyrus (DG) and eEF2K expression in SD rats

**Fig. 2:**
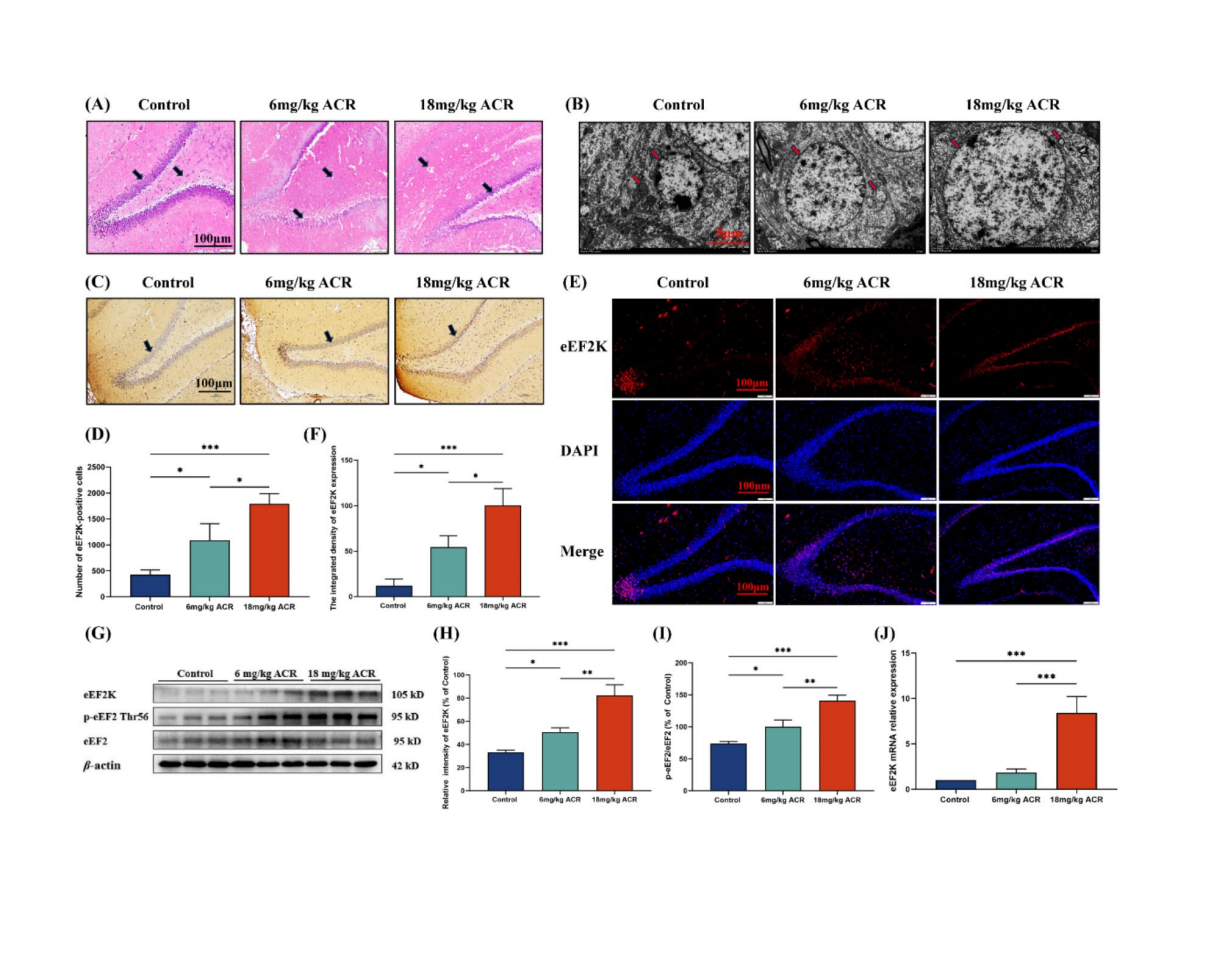
Effects of ACR on DG area and eEF2K expression in hippocampal tissues of SD rats. **(A)** HE staining to observe the pathological condition of the hippocampal DG areas. Black arrows indicate the morphology and neurons of the DG areas. Take pictures under a 200× light microscope, Scale bar:100 μm. **(B)** Transmission electron microscopy was used to observe changes in neurons in the hippocampal DG areas. Red arrows indicate neuronal morphology, mitochondrial morphology and liposomes. scale bar: 5 μm. **(C)** Immunohistochemistry was used to observe the expression of eEF2K in the hippocampal DG areas. Blue is the nucleus, and brown or tan is eEF2K. Black arrows indicate eEF2K-positive cells. Take pictures under a 200× light microscope, scale bar: 100 μm. **(D)** Expression number of eEF2K positive cells in the hippocampal DG areas. Data are expressed as means ± SD, *n* = 3. ^*^P < 0.05, ^***^P < 0.001. **(E)** Immunofluorescence method was used to observe the expression of eEF2K in the hippocampal DG areas. Red fluorescence represents eEF2K, and blue fluorescence represents the nucleus. scale bar: 100 μm. **(F)** Fluorescence intensity of eEF2K expression in the hippocampal DG areas. Data are expressed as means ± SD, *n* = 3. ^*^P < 0.05, ^***^P < 0.001. **(G)** Effects of ACR on the expression of eEF2, eEF2K, and p-eEF2 proteins in the hippocampus tissues of SD rats. **(H)** The protein expression levels of eEF2K in the hippocampus tissues of SD rats. Data are expressed as means ± SD, *n* = 3. ^*^P < 0.05, ^**^P < 0.01, ^***^P < 0.001. **(I)** Expression levels of p-eEF2/eEF2 in the hippocampus tissues of SD rats. Data are expressed as means ± SD, *n* = 3. ^*^P < 0.05, ^**^P < 0.01, ^***^P < 0.001. **(J)** Expression levels of eEF2K mRNA in hippocampal tissues. Data are expressed as means ± SD, *n* = 3. ^***^P < 0.001

The daily weight results of SD rats exposed to ACR showed that, compared to the control group, the rats exhibited slower weight gain with increasing ACR exposure concentration and longer exposure time (Supplementary Fig. 2A). The outcomes of the open field experiment revealed that rats in the high-dose ACR group were characterized by reduced time spent in the peripheral grid and an increase in time spent in the central grid, in comparison to the control group (*P* < 0.05) (Supplementary Fig. 2B, Supplementary Fig. 2C and Supplementary Fig. 2D). HE staining demonstrated morphological changes in the DG region of rat hippocampal tissue when compared with the control group. These changes included neuronal nuclear condensation and shrinkage, along with a gradual reduction in the number of neurons (Fig. 2A). Transmission electron microscopy observations revealed that, in contrast to the control group, neurons in the DG region of the hippocampal tissue from rats exposed to escalating ACR concentrations exhibited characteristics such as shrinkage, mitochondrial cristae rupture, cristae disappearance, increased vacuolization, and perinuclear edema (Fig. 2B).

Immunohistochemistry findings indicated that, relative to the control group, there was a significant increase in the number of eEF2K-positive cells in the DG region of the hippocampal tissue with escalating concentrations of ACR exposure (*P* < 0.05) (Fig. 2C and D). Immunofluorescence analysis revealed an elevated fluorescence intensity of eEF2K expression in the DG region of hippocampal tissue with increasing concentrations of ACR exposure, in contrast to the control group (*P* < 0.05) (Fig. 2E and F). Both Western blot and RT-qPCR analyses demonstrated that, in comparison to the control group, there was a dose-dependent increase in the protein expression levels of eEF2K and the ratio of p-eEF2/eEF2, along with an elevation in eEF2K mRNA expression with escalating concentrations of ACR exposure (*P* < 0.05) (Fig. 2G, H, I and J).

### Reducing eEF2K expression can improve the neurotoxicity of ACR on PC12 cells

**Fig. 3:**
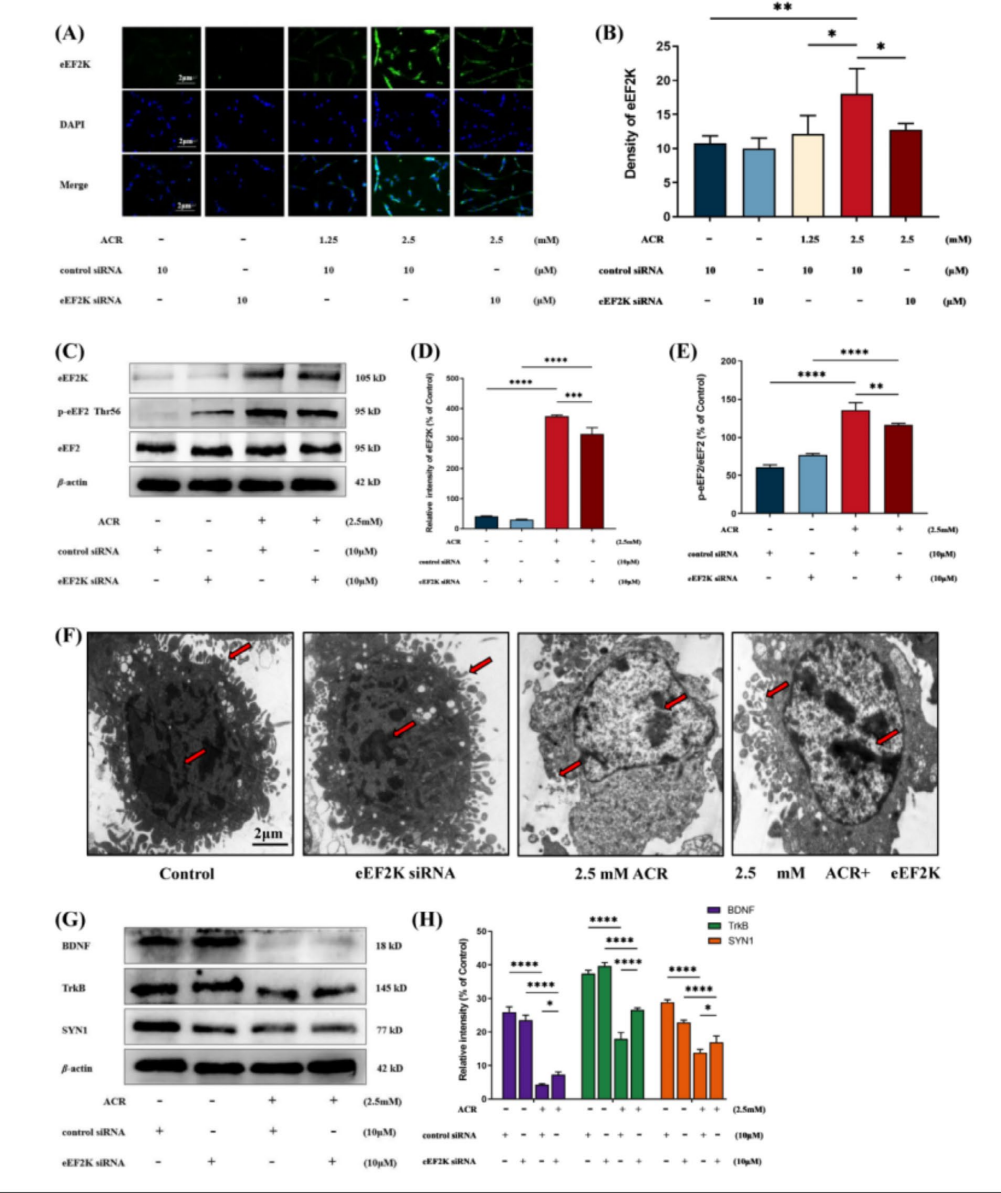
Administration of eEF2K siRNA in PC12 cells ameliorates neurotoxicity caused by ACR exposure. **(A)** Immunofluorescence observation of the fluorescent expression of eEF2K in different groups. Green fluorescence represents eEF2K, and blue fluorescence represents the nucleus. scale bar: 2 μm. **(B)** Fluorescence intensity of eEF2K in different groups. Data are expressed as means ± SD, *n* = 3. ^*^P < 0.05, ^**^P < 0.01. **(C)** Effects of ACR on the expression of eEF2, eEF2K and p-eEF2 proteins in different groups of PC12 cells. **(D)** Protein expression levels of eEF2K in different groups. Data are expressed as means ± SD, *n* = 3. ^***^P < 0.001, ^****^P < 0.0001. **(E)** Protein expression levels of p-eEF2/eEF2 in different groups. Data are expressed as means ± SD, *n* = 3. ^**^P < 0.01, ^****^P < 0.0001. **(F)** Morphological changes of PC12 cells in different groups were observed under transmission electron microscope. Red arrows indicate nuclear morphology as well as neurites in different groupings. scale bar: 2 μm. **(G)** Effects of ACR on the expression of learning and memory-related proteins BDNF, TrkB, and SYN1 in PC12 cells in different groups. **(H)** Protein expression levels of learning and memory-related proteins BDNF, TrkB, and SYN1 in different groups. Data are expressed as means ± SD, *n* = 3. ^*^P < 0.05, ^****^P < 0.0001

We used eEF2K-siRNA to inhibit eEF2K expression in low PC12 cells. CCK-8 experimental results showed that the cell survival rate of the group exposed to ACR after eEF2K-siRNA interference was higher than that of the PC12 cell group exposed to ACR alone (*P* < 0.05) (Supplementary Fig. 3). Immunofluorescence results showed that at the same concentration of ACR (2.5 mM), the eEF2K fluorescence expression intensity of cells exposed to ACR after eEF2K-siRNA interference was significantly lower than that of cells exposed to ACR alone (Fig. 3A and B). Western blot experiments showed that, at the same exposure concentration of ACR (2.5 mM), the protein expression of eEF2K and p-eEF2/eEF2 in the ACR group after eEF2K-siRNA interference was significantly lower than that in the cells exposed to the ACR group alone (Fig. 3C, D and E). Transmission electron microscopy results showed that, compared with the control group, the ACR exposure group exhibited nuclear condensation, absence of neurite outgrowth, mitochondrial swelling, and vacuolation. In contrast, the cells in the group where eEF2K expression was inhibited by siRNA and then exposed to ACR showed relatively intact nuclei, increased neurite outgrowth, mild mitochondrial swelling, and vacuolation (Fig. 3F). We measured the expression of proteins related to learning and memory (BDNF, TrkB and SYN1) in cells. The Western blot results indicate that, compared to the group exposed solely to ACR (2.5 mM), the group exposed to ACR (2.5 mM) following eEF2K-siRNA interference shows a significantly higher expression level of learning and memory-related proteins (BDNF, TrkB, and SYN1) (*P* < 0.05) (Fig. 3G and H).

### eEF2K-KO mice can improve behavioral performance related to learning and memory caused by ACR

**Fig. 4:**
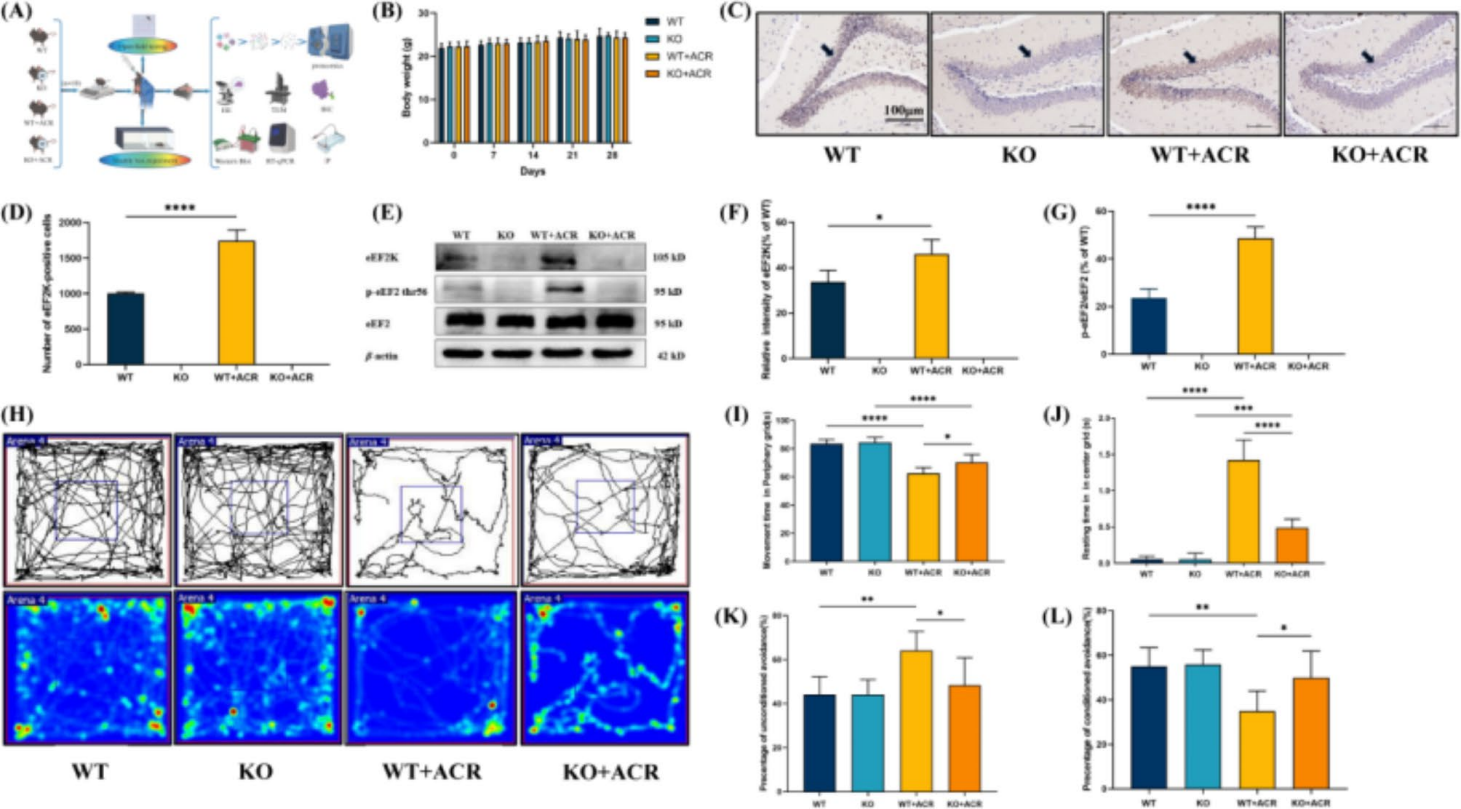
eEF2K-KO mice can improve behavioral performance related to learning and memory impairment caused by ACR exposure. **(A)** Flowchart of experiments conducted on eEF2K-KO mice. **(B)** Body weight changes in eEF2K-KO mice exposed to ACR for 28 days. **(C)** Immunohistochemistry was used to observe the expression of eEF2K in the DG area of hippocampal tissue. Blue is the nucleus, and brown or tan is eEF2K. Black arrows indicate the positive expression of eEF2K. Take pictures under a 200× light microscope, scale bar: 100 μm. **(D)** Expression of eEF2K-positive cells in the DG area of hippocampal tissue. Data are expressed as means ± SD, *n* = 3. ^****^P < 0.0001. **(E)** Effects of ACR on eEF2, eEF2K, and p-eEF2 protein expression in eEF2K-KO mice. **(F)** The protein expression intensity of eEF2K in the hippocampus tissue of eEF2K-KO mice. Data are expressed as means ± SD, *n* = 3. ^*^P < 0.05. **(G)** The protein expression intensity of p-eEF2/eEF2 in the hippocampus tissue of eEF2K-KO mice. Data are expressed as means ± SD, *n* = 3. ^****^P < 0.0001. **(H)** Path diagram and heat map of open field testing. **(I)** Peripheral grid movement time in the open field test. Data are expressed as means ± SD, *n* = 6. ^*^P < 0.05, ^****^P < 0.0001. **(J)** Centre grid resting time in the open field test. Data are expressed as means ± SD, *n* = 6. ^***^P < 0.001; ^****^P < 0.0001. **(K)** Percentage of unconditioned responses in shuttle box test. Data are expressed as means ± SD, *n* = 6. ^*^P < 0.05; ^**^P < 0.01. **(L)** Percentage of conditioned responses in shuttle box test. Data are expressed as means ± SD, *n* = 6. ^*^P < 0.05; ^**^P < 0.01

eEF2K^−/−^ (KO) mice were generated by targeting exons 7–10 of the eEF2K-201 (ENSMUST00000047875.15) transcript as the knockout region, with only 224 bp of DNA remaining in the KO mice. The 224 bp band is observed in PCR①, but not in PCR②, indicating the presence of KO mice. The PCR analysis revealed a 4969 bp band in PCR① and a 270 bp band in PCR②, indicating the presence of the wild-type (WT) mouse (Supplementary Fig. 4). We presented the experimental design in mouse studies (Fig. 4A). The weight results demonstrated that WT + ACR and KO + ACR mice exhibited a slower weight gain compared to WT and KO mice (Fig. 4B). Immunohistochemistry results showed a significant increase in the number of eEF2K-positive cells in the DG of WT + ACR mice compared to WT mice (Fig. 4C and D). Western blot analysis demonstrated a notable elevation in the expression levels of eEF2K and the ratio of p-eEF2/eEF2 proteins in the hippocampus of WT + ACR mice in comparison to WT mice (Fig. 4E, F and G).

Behavioral test results showed obvious differences in the behavior of mice in different groups. In the open field experimental test, compared with the mice in the WT group, the mice in the WT + ACR group mainly showed a significant decrease in the movement time of the surrounding grid and the significant increase in the residence time of the central grid. However, mice in the KO + ACR group mainly manifested a significantly increased movement time in the peripheral grid and shortened stay time in the central grid (*P* < 0.05) (Fig. 4H, I and J). The shuttle box experiment results revealed that, relative to mice in the WT group, the WT + ACR group exhibited a notable increase in the percentage of unconditioned responses, accompanied by a significant decrease in the percentage of conditioned responses. In contrast, when compared with mice in the WT + ACR group, mice in the KO + ACR group had a relatively lower percentage of unconditioned responses and a relatively higher percentage of conditioned responses (*P* < 0.05) (Fig. 4K and L).

### eEF2K-KO mice can improve learning and memory-related indicators caused by ACR

**Fig. 5:**
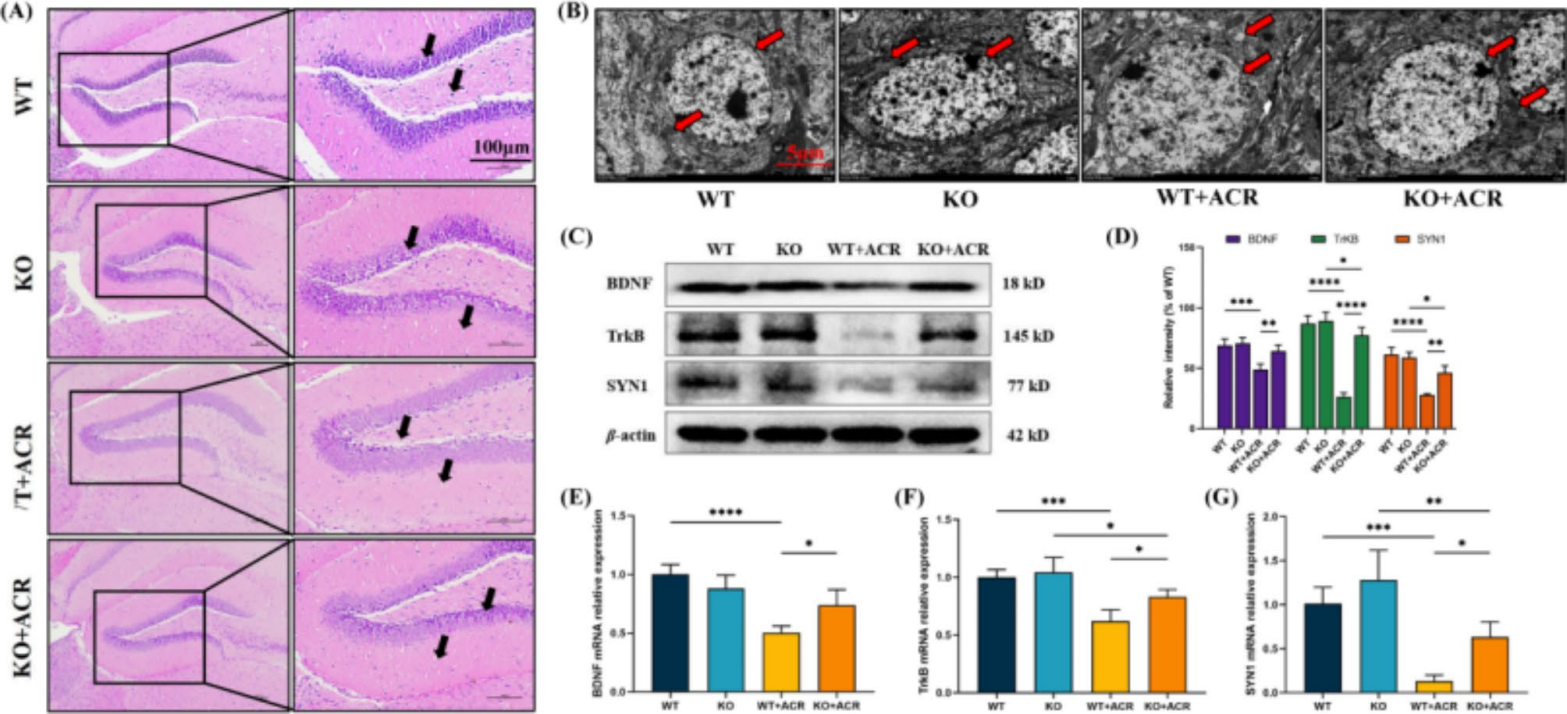
eEF2K-KO mice can improve learning and memory impairment caused by ACR exposure. **(A)** HE staining was used to observe the pathological conditions of the DG area of hippocampal tissue in different groups. The black arrow in the figure indicates the morphology and neurons of the DG area. The picture on the right was taken under a 200× optical microscope. Scale bar: 100 μm. **(B)** Transmission electron microscopy was used to observe changes in neurons in the DG area of hippocampal tissue in eEF2K-KO mice after exposure to ACR. Red arrows indicate neuronal and mitochondrial morphology. scale bar: 5 μm. **(C)** The effect of ACR on the expression of learning and memory-related proteins BDNF, TrkB and SYN1 in different groups. **(D)** Protein expression intensity of learning and memory-related proteins BDNF, TrkB, and SYN1 in different groups. Data are expressed as means ± SD, *n* = 3. ^*^P < 0.05, ^**^P < 0.01, ^***^P < 0.001, ^****^P < 0.0001. **(E) (F) (G)** BDNF mRNA, TrkB mRNA and SYN1 mRNA expression intensity in different groups. Data are expressed as means ± SD, *n* = 4. ^*^P < 0.05, ^**^P < 0.01, ^***^P < 0.001, ^****^P < 0.0001

HE staining outcomes indicated that, in contrast to mice in the WT group and the KO group, the DG area of the hippocampus in the WT + ACR group displayed evident nuclear condensation and shrinkage. However, the nuclear condensation and shrinkage phenomena in the DG area of the hippocampal tissue of mice in the KO + ACR group were improved (Fig. 5A). Transmission electron microscopy findings revealed that, relative to the DG area of the hippocampal tissue in mice from the WT group and KO group, neurons in the DG area of the hippocampal tissue in the WT + ACR group exhibited swelling, along with mitochondrial cristae breakage, cristae disappearance, and slurry edema. However, the neurons in the DG area of the hippocampus of mice in the KO + ACR group only showed mild swelling, and the mitochondrial morphology was intact (Fig. 5B).

Western blot results illustrated that, in relation to mice in the WT + ACR group, the protein expression levels of BDNF, TrkB and SYN1 in the hippocampus of mice in the KO + ACR group were significantly elevated (*P* < 0.05) (Fig. 5C and D). Additionally, RT-qPCR findings demonstrated that, compared to mice in the WT + ACR group, the mRNA expression levels of BDNF, TrkB and SYN1 in the hippocampus of mice in the KO + ACR group were significantly increased (*P* < 0.05) (Fig. 5E, F and G).

### The possible mechanism by which eEF2K plays a role in the impairment of learning and memory caused by ACR is by affecting ether lipid metabolism

**Fig. 6:**
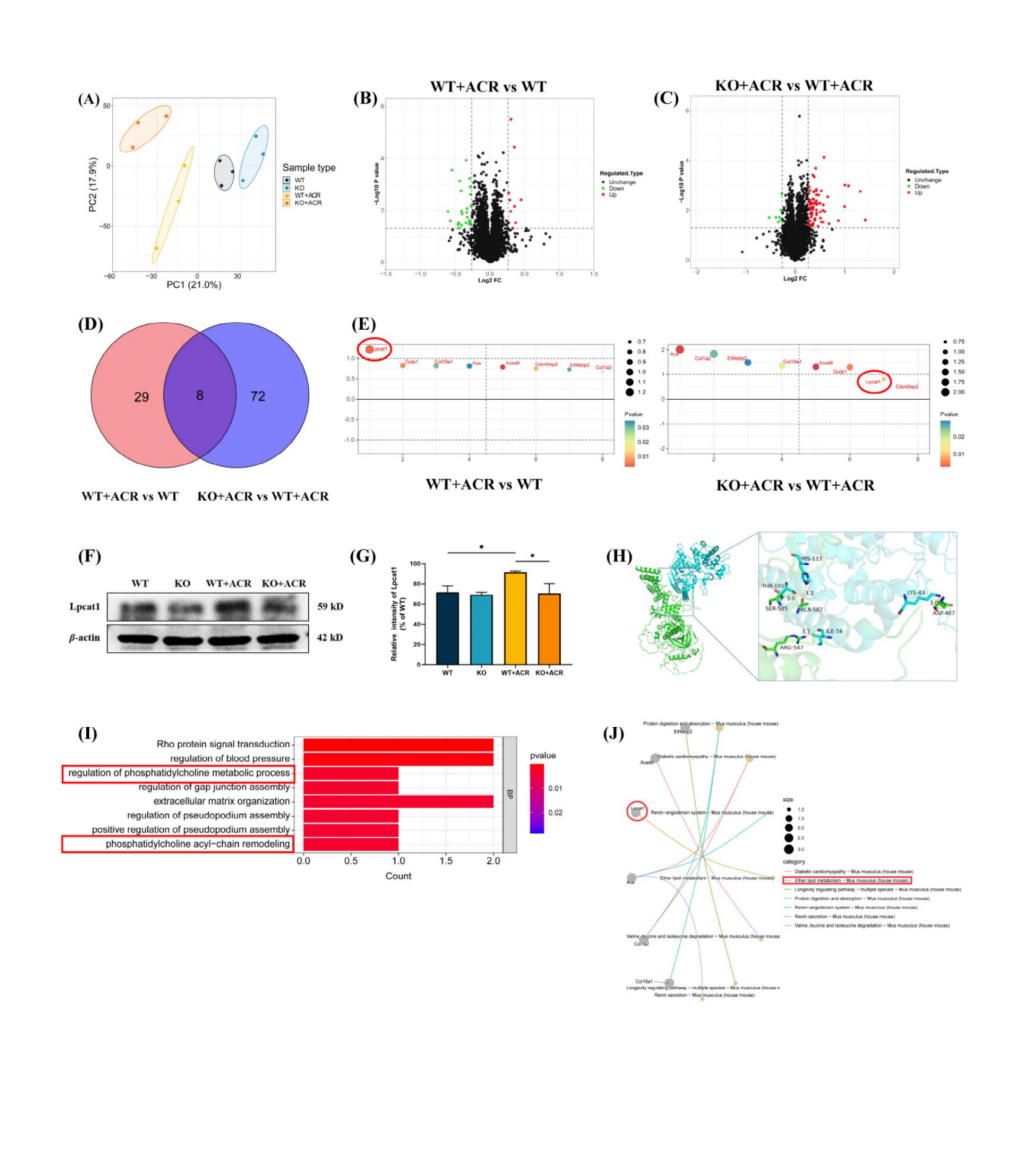
eEF2K plays a role in the learning and memory impairment caused by ACR by affecting ether lipid metabolism. **(A)** The WT group, KO group, WT + ACR group and KO + ACR group are completely distinguished by different colors in the PCA plot. **(B)** Volcano plot of differentially expressed proteins between WT group and WT + ACR group. Fold change ≥ 1.2, *P* < 0.05. **(C)** Volcano plot of differentially expressed proteins between KO + ACR group and WT + ACR group. Fold change ≥ 1.2, *P* < 0.05. **(D)** Venn plot screened 8 differential proteins that were only regulated by eEF2K. **(E)** Perform protein ranking dot plot analysis on the screened 8 proteins. The expression of Lpcat1 is the most significant. **(F)** Effect of ACR on Lpcat1 expression in hippocampus tissue of eEF2K-KO mice. **(G)** Lpcat1 protein expression intensity in different groups. Data are expressed as means ± SD, *n* = 3. ^*^P < 0.05. **(H)** Protein-protein docking of eEF2K to its downstream Lpcat1. Green ribbon structure: eEF2K, blue ribbon structure: Lpcat1. Rod-like structure: interacting amino acid residues. Yellow dashed line: hydrogen bond. Red dashed line: hydrophobic interaction, green dashed line: hydrogen bond interaction. **(I)** The top 8 major BPs analyzed by GO enrichment. Lpcat1 participates in 2 major BPs. **(J)** KEGG pathway analysis showed that Lpcat1 mainly regulates the ether lipid metabolism pathway

Principal component analysis (PCA) results showed that the 12 samples were significantly divided into four groups (Fig. 6A). Pearson correlation between differential protein analysis samples showed that there was a similar relationship between the 12 sequencing samples (Supplementary Fig. 5). Compared with the WT group, there were 37 differential proteins in the WT + ACR group, including 10 upregulated proteins and 27 downregulated proteins (Fig. 6B). Compared with the WT + ACR group, there were 80 differential proteins in the KO + ACR group, including 73 upregulated proteins and 7 downregulated proteins (Fig. 6C). The Venn diagram results indicate that there are 8 differentially expressed proteins regulated by eEF2K (Fold change ≥ 1.2, *P* < 0.05). These proteins include Lpcat1, Ace, Col18a1, Col1a2, Gulp1, Acad8, Eif4ebp2, and Cdc42ep2 (Supplementary Table 3 and Fig. 6D). The protein ranking dot plot results showed that Lpcat1 was the only differential protein that was upregulated in the WT + ACR group compared with the WT group and downregulated in the KO + ACR group compared with the WT + ACR group (Fig. 6E).

Western blot results showed that, compared with mice in the WT + ACR group, Lpcat1 protein expression in the hippocampus of mice in the KO + ACR group was significantly reduced (*P* < 0.05) (Fig. 6F and G). The protein docking results between eEF2K and Lpcat1 indicate that an intermolecular interaction can be formed between the two. The binding score between the two proteins was − 266.41, with a confidence score of 91%. The binding affinity of the two proteins is relatively strong, indicating that Lpcat1 may be a downstream protein of eEF2K (Fig. 6H). The enrichment analysis of the GO database identified the top 8 key biological processes (BPs), among which Lpcat1 was found to be involved in 2 major BPs, namely, regulation of phosphatidylcholine metabolic process and phosphatidylcholine acyl chain remodeling (Fig. 6I). KEGG pathway analysis results showed that the main pathway regulated by Lpcat1 is Ether lipid metabolism (Fig. 6J).

## Discussion

The impairment of learning and memory caused by ACR has garnered attention from scholars worldwide. ACR has been found to cause learning and memory impairment in vivo, in vitro, and epidemiological studies [32, 33]. However, little is known about research on ACR-induced learning and memory impairment, particularly among the population occupationally exposed to ACR, which could serve as a breakthrough in understanding this phenomenon. In the present study, the ACR occupationally exposed groups that we investigated all had more than 10 years of working experience. In the subjective symptom survey, we found that the ACR exposure group had a higher incidence of subjective symptoms related to learning and memory than the non-exposed group (*P* < 0.05). Studies demonstrated that tunnel workers exposed to ACR experienced memory impairment [34], which is consistent with the results of our study. These findings indicate that long-term low-dose exposure to ACR may cause learning and memory impairment.

The potential mechanisms through which ACR causes learning and memory impairment include neuronal damage, inflammatory response, neurotransmitter imbalance, oxidative stress, and release of neuroinflammatory factors, etc. [32, 33, 35, 36]. However, the important targets of ACR that cause learning and memory impairment, and the specific damage mechanism are still unclear. In the present study, a serum proteomic investigation was undertaken in populations occupationally exposed to ACR to identify key target proteins associated with ACR-induced learning and memory impairment. Our results show that in addition to proteins related to the currently known mechanism of ACR induced learning and memory impairment, the most significantly upregulated protein was elongation factor 2 (eEF2). The primary role of eukaryotic elongation factor 2 (eEF2) is to participate in the elongation phase of protein synthesis, with the phosphorylation of eEF2 representing a key mechanism in this phase. Eukaryotic elongation factor 2 kinase (eEF2K) is the exclusive protein kinase known to phosphorylate eEF2 at the 56th threonine residue (Thr-56, p-eEF2) [37]. Studies indicate that eEF2K is expressed in neurons and may contribute to processes such as learning, memory, and depression [38, 39]. Given the crucial role of new protein synthesis in long-term memory, eEF2K presents a novel research avenue for investigating learning and memory impairment in brain neurons.

A recent study shows that suppressing the eEF2K mRNA translation factor alleviates learning and memory impairments in aged mice [39]. Research has shown that increased activity of eEF2K is found in the hippocampus of deceased patients with Alzheimer’s disease, and inhibiting the activity of eEF2K can prevent the toxic effects of Aβ42 oligomers on neurons [40]. Therefore, investigating eEF2K proves beneficial in uncovering the pathogenesis of prominent neurodegenerative diseases, including Alzheimer’s disease and Parkinson’s disease. However, it is still unclear whether eEF2K is involved in the process of learning and memory impairment caused by ACR. Our results showed that as the ACR exposure concentration increased, the expression of eEF2K in rat hippocampus tissue and PC12 cells also increased. Reducing the expression of eEF2K in PC12 cells can effectively improve the expression of learning and memory-related proteins after ACR exposure. These findings suggest that eEF2K may be involved in the process of learning and memory impairment caused by ACR.

Research shows that eEF2K-KO mice can show good life characteristics and have normal cognitive behaviors [41–44]. Recent studies suggest that eEF2K-KO mice exhibit alleviation of age-related long-term recognition memory impairment [45, 46]. Therefore, we used eEF2K-KO mice to explore whether eEF2K is an important target in the process of learning and memory impairment caused by ACR. The results of this study demonstrated that, compared to the WT + ACR group, the KO + ACR group of mice showed significant improvements in behavioral tests related to learning and memory, pathological changes in the hippocampal tissue (DG), and the expression of proteins related to learning and memory. This suggests that eEF2K has a hub function in the process of learning and memory impairment caused by ACR and is an important target protein.

In order to further understand the role and mechanism of eEF2K in the process of learning and memory impairment caused by ACR, we conducted proteomics analysis on mouse hippocampal tissue. Our results showed that an important downstream protein affected by eEF2K is Lpcat1 and mainly affects the ether lipid metabolism pathway. Ether lipids are a specific class of synaptic lipids that are particularly important for synaptic vesicle recycling due to their unique biophysical properties [47]. Studies have shown that the lack of ether lipids can directly affect the endocytosis and exocytosis processes of synaptic vesicle recycling [48]. Synaptic vesicles are rich in phospholipids, especially phosphatidylcholine and phosphatidylethanolamine. These phospholipids are involved in neurotransmitter release and synaptic transmission by regulating the formation, transport, and release of synaptic vesicles, which are closely related to ether lipid metabolism [49]. Research indicates a close correlation between synaptic vesicles and synaptic plasticity, collectively playing a crucial role in maintaining the functionality of normal learning and memory [50]. Current research on the impact of eEF2K on learning and memory has consistently shown that eEF2K may lead to learning, memory and cognitive dysfunction by changing synaptic plasticity and/or the balance between excitation and inhibition [51, 52]. This indicates that eEF2K affects ether lipid metabolism through Lpcat1, thereby playing a role in the process of learning and memory impairment caused by ACR.

Our research has the following advantages. Firstly, we conducted a study for the first time using the ACR occupationally exposed population as the starting point, and utilized proteomics technology to identify important target proteins associated with ACR-induced learning and memory impairment. Additionally, we proposed for the first time that eEF2K can serve as an important target in ACR-induced learning and memory impairment. We validated the role of eEF2K in the process of ACR-induced learning and memory impairment through both in vitro and in vivo experiments, and elucidated the possible mechanisms of action. Furthermore, eEF2K is widely used as a target protein in cancer research, so our study represents a novel application of an old target, breaking through the limitations of scientific research.

Our study also has certain limitations that need to be addressed in future research. Further investigation is needed to validate the crucial role of intervening eEF2K expression in the process of ACR-induced learning and memory impairment by conducting local eEF2K knockout in mouse brains. Additionally, we did not further explore the specific interaction between eEF2K and ether lipid metabolism in this study. Therefore, it is still necessary to intervene in the population in subsequent studies and further confirm the target effect of eEF2K in clinical experiments.

## Conclusion

In summary, we have employed multiple approaches to fully demonstrate the target role of eEF2K in the process of learning and memory impairment caused by ACR, and further innovatively elucidated its mechanism of action, filling a gap in the research field. As eEF2K is a small molecule kinase, the development of inhibitors targeting eEF2K may become a novel strategy for the prevention or treatment of ACR-induced learning and memory impairment. Additionally, the elucidation of the function and regulatory mechanism of eEF2K in this study contributes to a deeper understanding of the pathogenesis of central nervous system diseases, providing a solid theoretical basis for future research on the occurrence of learning and memory impairment caused by ACR.

### Electronic supplementary material

Below is the link to the electronic supplementary material.

Supplementary Material 1

Supplementary Material 2

Supplementary Material 3

Supplementary Material 4

Supplementary Material 5

Supplementary Material 6

Supplementary Material 7

Supplementary Material 8

Supplementary Material 9

Supplementary Material 10

Supplementary Material 11

Supplementary Material 12

Supplementary Material 13

Supplementary Material 14

Supplementary Material 15

Supplementary Material 16

Supplementary Material 17

Supplementary Material 18

Supplementary Material 19

## Data Availability

The original data presented in this study are included in the article/additional files, and further inquiries can be directed to the corresponding authors.
