# Supplementary material for: Reduction of eEF2 kinase alleviates the learning and memory impairment caused by acrylamide
Source: Cell Biosci. 2024 Aug 23;14:106. doi: 10.1186/s13578-024-01285-7 (PMC11344312; doi:10.1186/s13578-024-01285-7)
Supplement: Supplementary file 1 — Supplementary Material 1 [file 13578_2024_1285_MOESM1_ESM.docx]

**Highlights**

Proteomic analysis of serum in populations occupationally exposed to ACR was conducted.

eEF2K is associated with impairment of learning and memory caused by ACR exposure.

Knocking down eEF2K can improve learning and memory impairment caused by ACR.

eEF2K influences learning and memory functions through ether lipid metabolism.

eEF2K is a key target for preventing and treating ACR-induced impairment of learning and memory.
