# Supplementary material for: Reduction of eEF2 kinase alleviates the learning and memory impairment caused by acrylamide
Source: Cell Biosci. 2024 Aug 23;14:106. doi: 10.1186/s13578-024-01285-7 (PMC11344312; doi:10.1186/s13578-024-01285-7)
Supplement: Supplementary file 2 — Supplementary Material 2 [file 13578_2024_1285_MOESM2_ESM.docx]

1. **
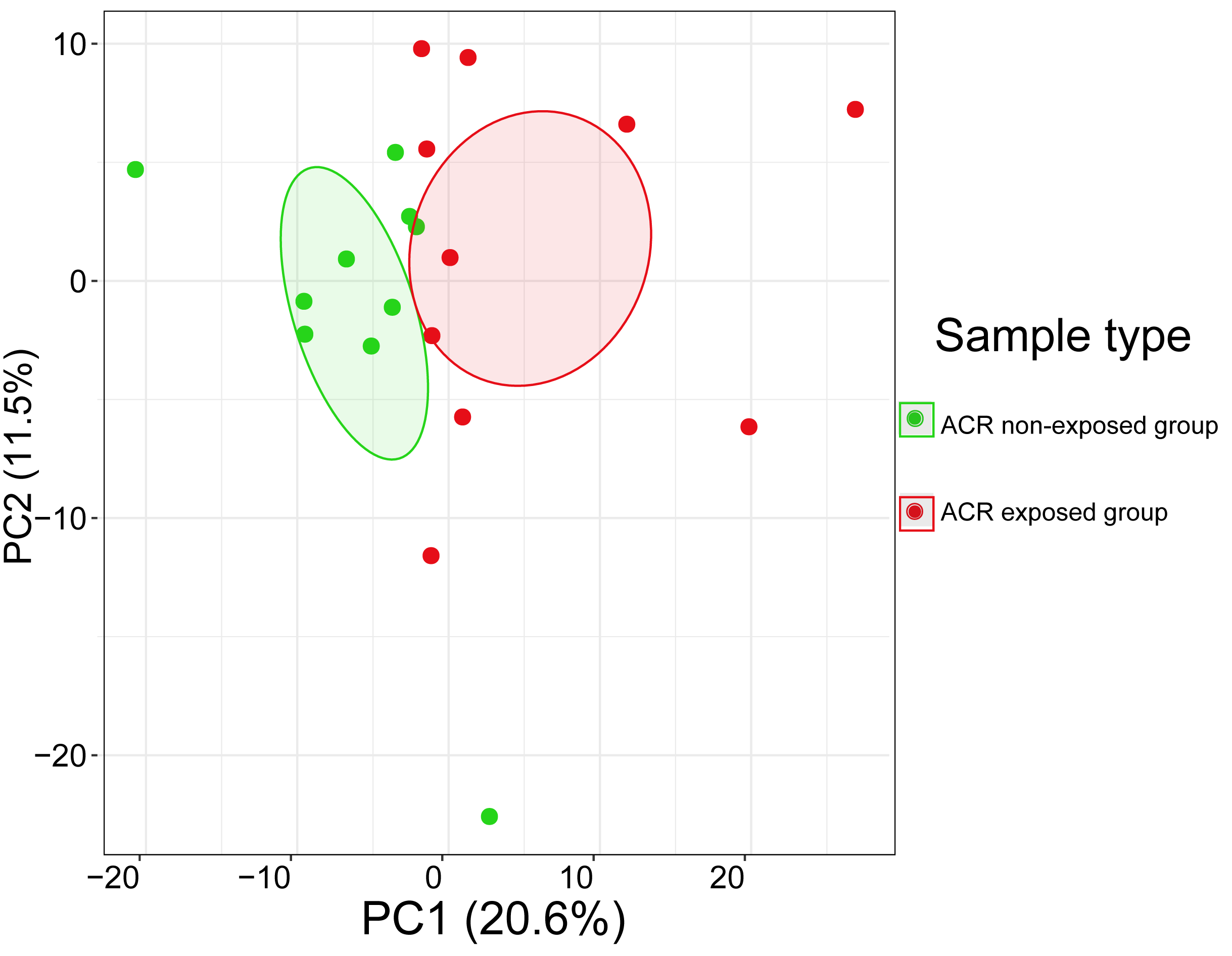
 (B)**

**
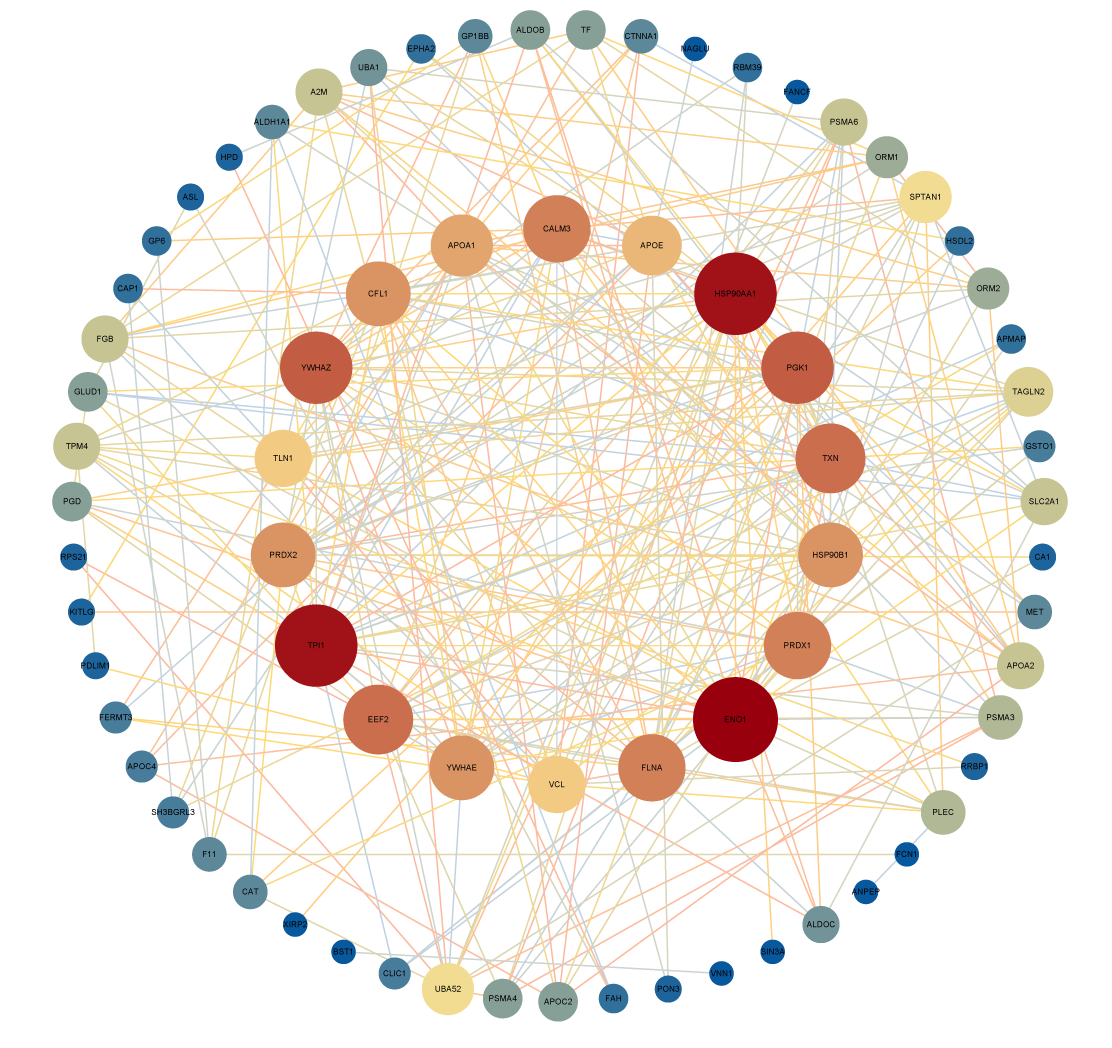

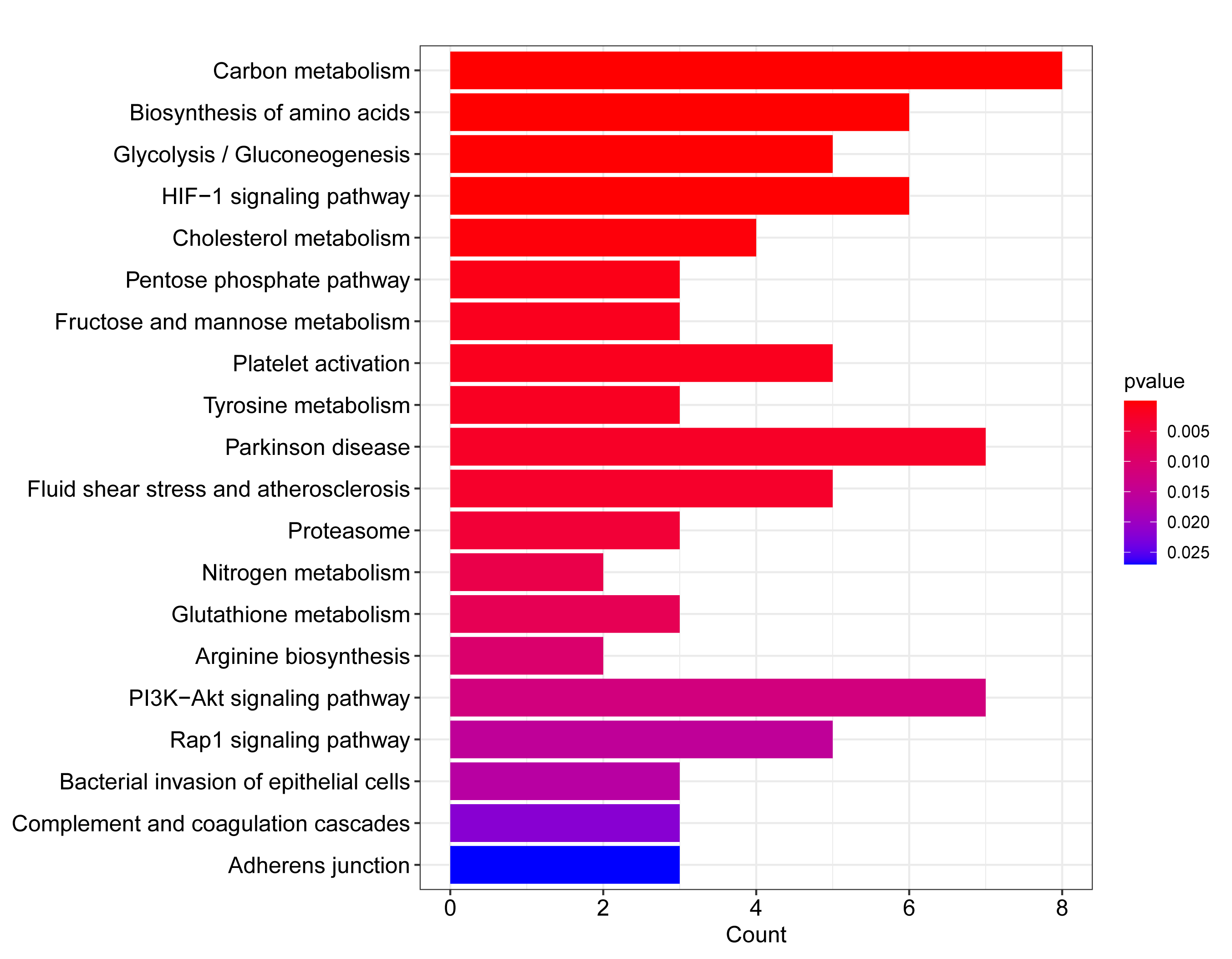
 (C) (D)**

**Supplementary Figure 1. Results of differential protein analysis of population serum proteomics.**

1. The ACR-exposed and non-exposed groups were completely distinguished in the PCA plot.
2. GO enrichment analysis diagram of differential proteins. *P*<0.05，*P* just =1，Fold change≥1.2.
3. KEGG pathway analysis diagram of differential proteins. *P*<0.05，*P* just =1，Fold change≥1.2.
4. Differential protein interaction network diagram.

**
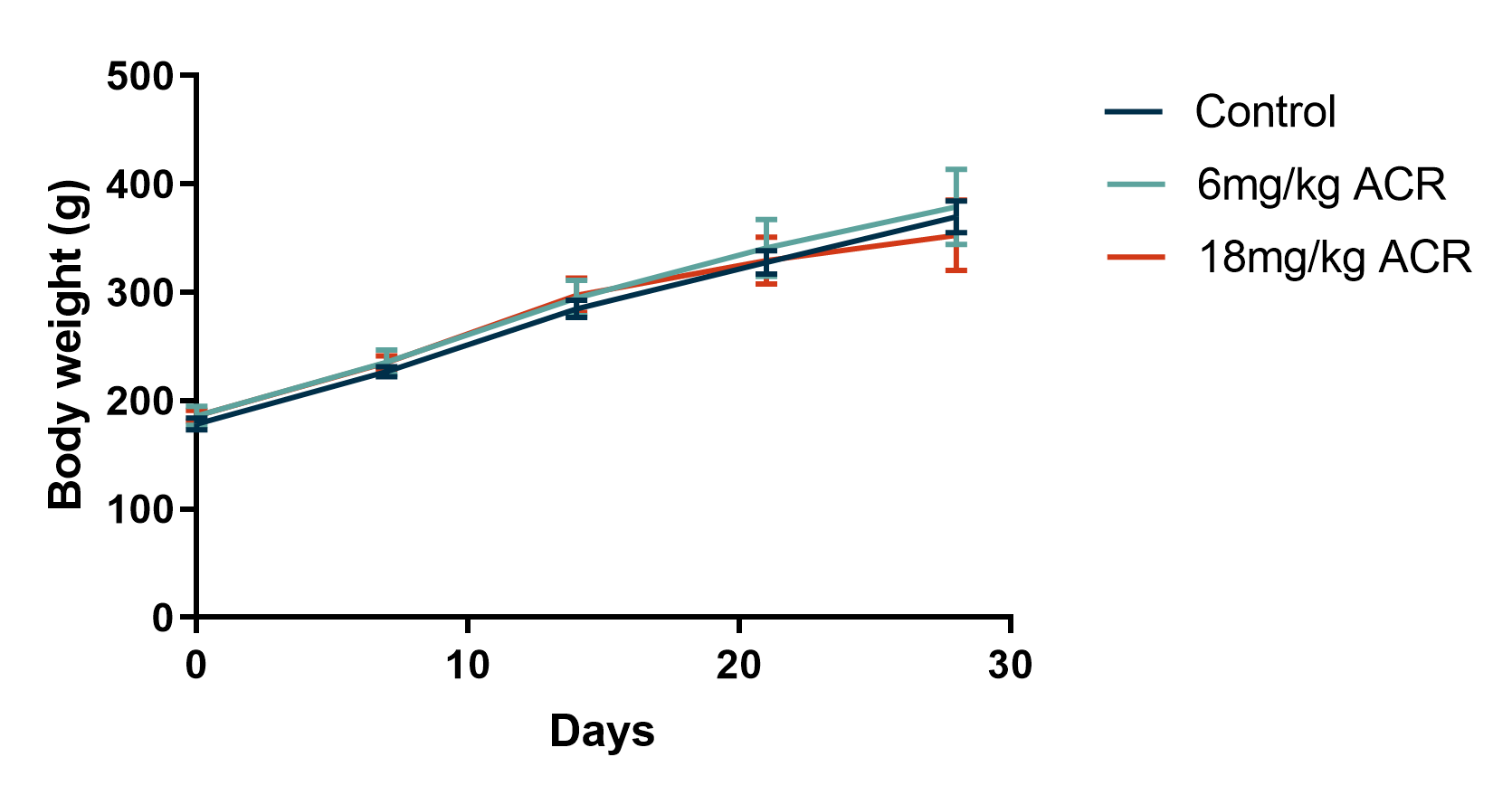
(A)**


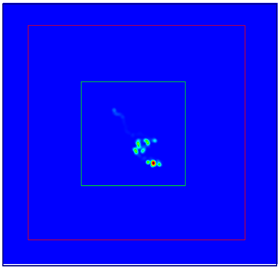

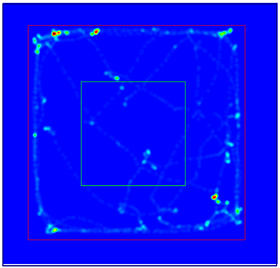

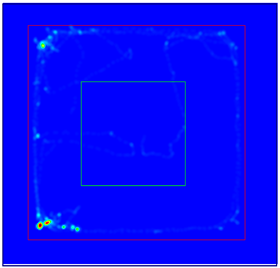
**(B)**

**Control**

**6mg/kg ACR**

**18mg/kg ACR**


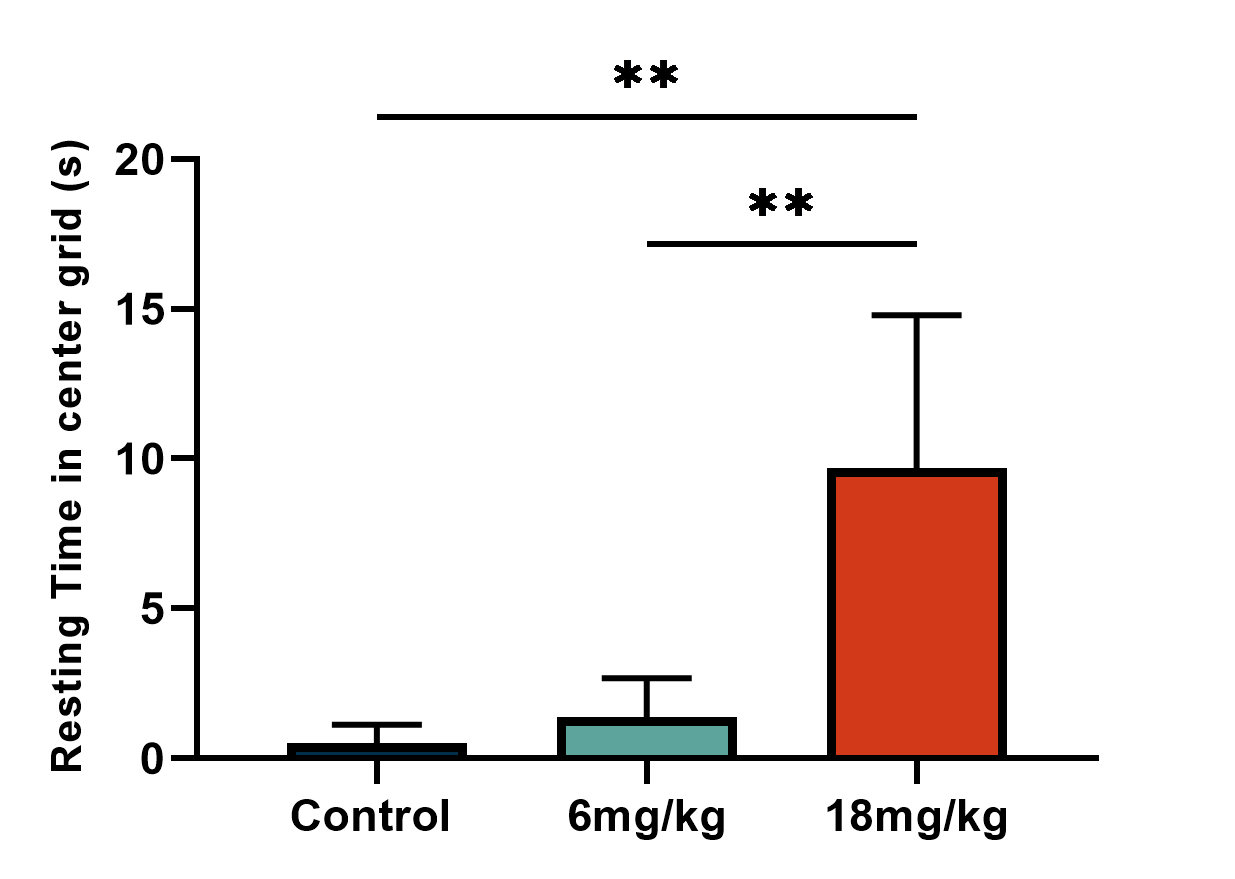

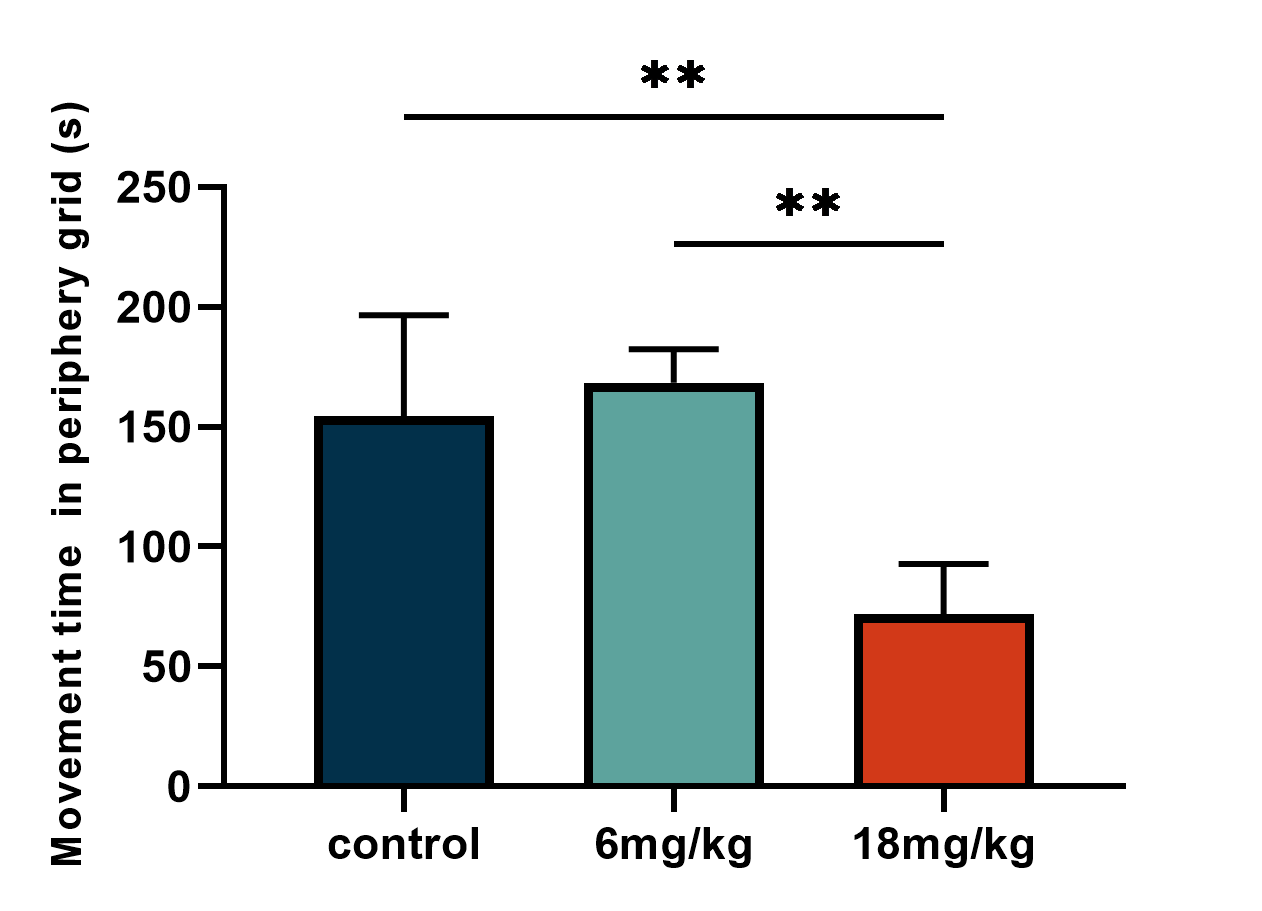


**(C) (D)**

**Supplementary Figure 2. Body weight recording and behavioral experiments of SD rats.**

1. Body weight changes in ACR-exposed SD rats within 28 days.
2. Heat map of open field test after ACR exposure in SD rats.
3. Periphery grid movement time in the open field test. Data are expressed as means ± SD, n=4. ^**^*P*<0.01.
4. Centre grid resting time in the open field test. Data are expressed as means ± SD, n=4. ^**^*P*<0.01.


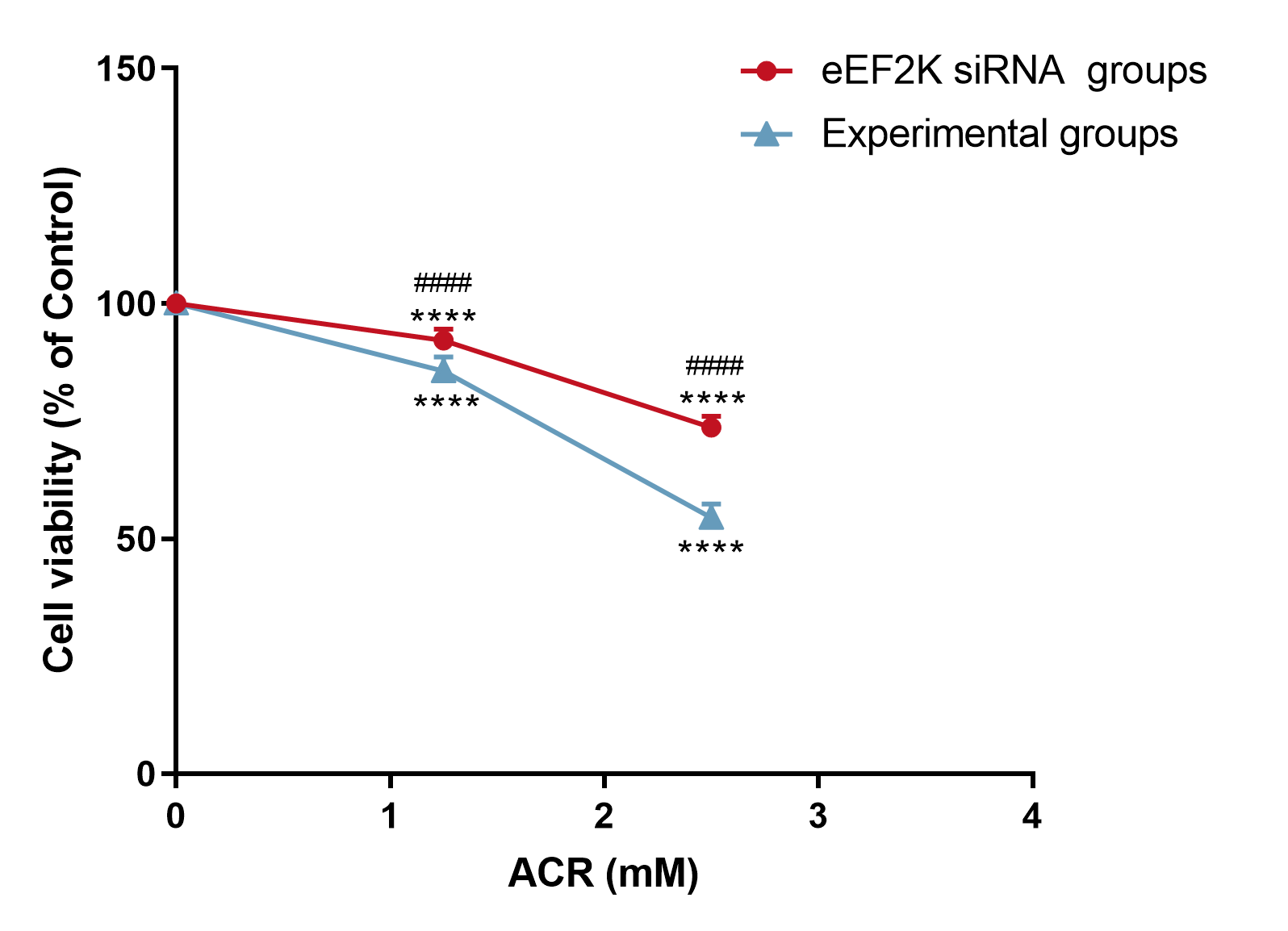


**Supplementary Figure 3. CCK-8 assay measures cell survival after ACR exposure of PC12 cells.**^****^*P*<0.0001 compared with the control group; ^####^*P*<0.0001 compared with the experimental group exposed to the same ACR concentration.

**
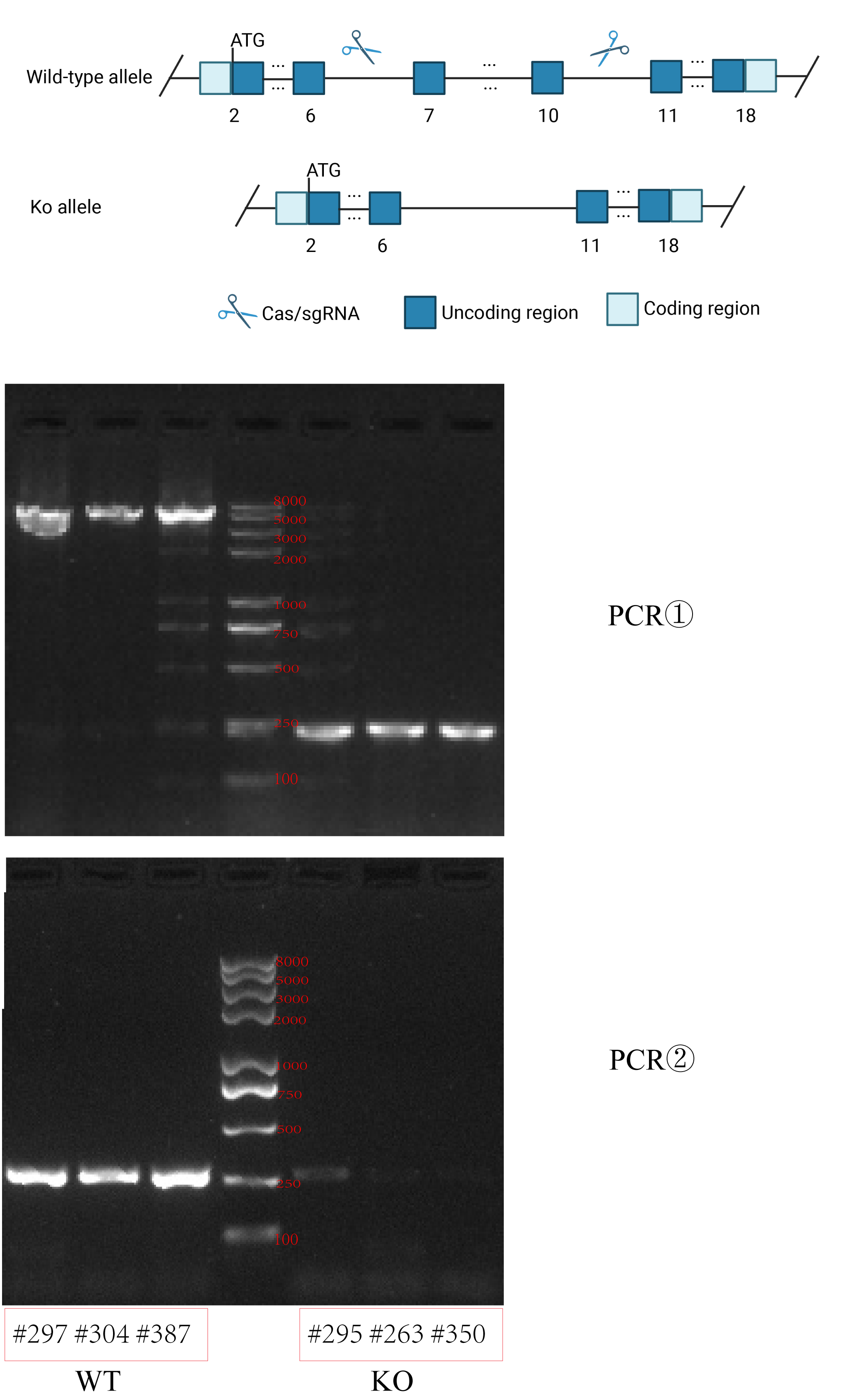
**

**Supplementary Figure 4. eEF2K-KO mouse gene knockout strategy.** The eEF2K gene was modified using CRISPR/Cas9 technology.

**
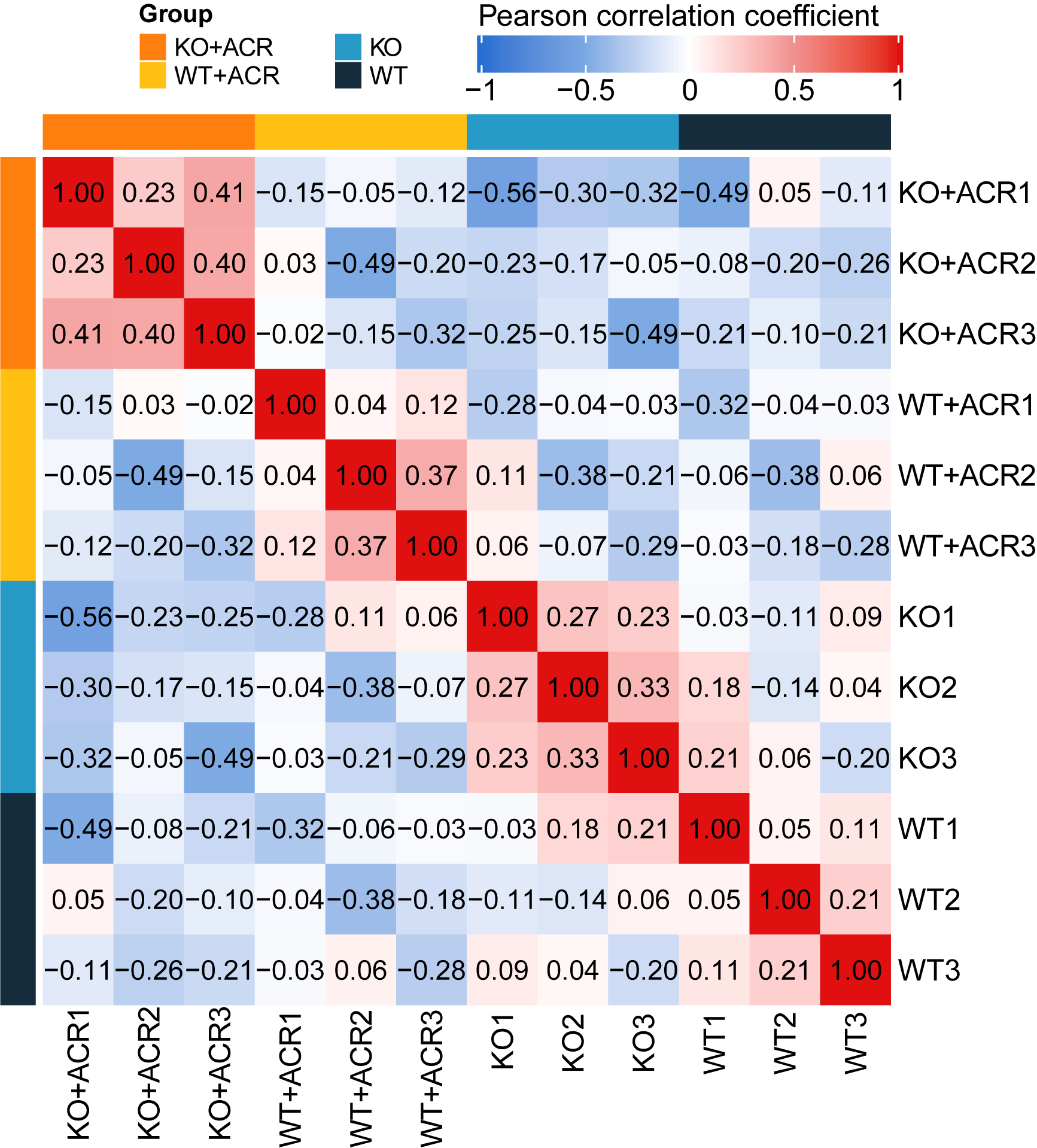
**

**Supplementary Figure 5. Pearson correlation analysis between samples from each group of eEF2K-KO mice.**
