# Supplementary material for: Reduction of eEF2 kinase alleviates the learning and memory impairment caused by acrylamide
Source: Cell Biosci. 2024 Aug 23;14:106. doi: 10.1186/s13578-024-01285-7 (PMC11344312; doi:10.1186/s13578-024-01285-7)
Supplement: Supplementary file 3 — Supplementary Material 3 [file 13578_2024_1285_MOESM3_ESM.docx]

**Liquid chromatography-mass spectrometry (LC-MS) analysis**

Peptides dissolved in mobile phase A were separated using an EASY-nLC 1200 ultra-high-performance liquid chromatography system. Mobile phase A consisted of a water solution containing 0.1% formic acid and 2% acetonitrile, while mobile phase B consisted of a water solution containing 0.1% formic acid and 90% acetonitrile. The liquid phase gradient was set as follows: 0-4 min, 7%~10% B; 4.0-53.0 min, 10%~32% B; 53.0-57.0 min, 32%~80% B; 57.0-60.0 min, 80% B, with a flow rate maintained at 500 nL/min. After separation by the ultra-high-performance liquid chromatography system, the peptides were ionized in the NSI ion source and then analyzed using the Orbitrap Exploris™ 480 mass spectrometer (ThermoFisher Scientific). The ion source voltage was set at 2.3 kV, and the FAIMS compensation voltage (CV) was set at -45 V. Both the parent ions and their secondary fragments were detected and analyzed using the high-resolution Orbitrap. The first mass spectrometry scan range was set at 400 - 1200 m/z, with a scan resolution of 60000. The second mass spectrometry scan range had a fixed starting point of 110 m/z, with a scan resolution of 15000. Turbo TMT was set as TMT Reagents.

Data acquisition was performed using a data-dependent scan (DDA) program, where the top 25 peptide parent ions with the highest signal intensity from the first scan were sequentially subjected to collision-induced dissociation (CID) in the HCD collision cell using 35% collision energy, followed by sequential analysis using second mass spectrometry. To improve the efficiency of mass spectrometry utilization, the automatic gain control (AGC) was set at 100%, the signal threshold was set at 5.0e4 ions/s, the maximum injection time was set to Auto, and the dynamic exclusion time for tandem mass spectrometry scans was set to 30 s to avoid repeated scanning of parent ions.
