# Supplementary material for: Reduction of eEF2 kinase alleviates the learning and memory impairment caused by acrylamide
Source: Cell Biosci. 2024 Aug 23;14:106. doi: 10.1186/s13578-024-01285-7 (PMC11344312; doi:10.1186/s13578-024-01285-7)
Supplement: Supplementary file 4 — Supplementary Material 4 [file 13578_2024_1285_MOESM4_ESM.docx]

**Molecular docking**

We utilized the HDOCK online platform (http://hdock.phys.hust.edu.cn/) as the molecular docking program for this study. The structures of the docking proteins eEF2K and Lpcat1, with UniProt protein database IDs O08796 and Q3TFD2 respectively, were downloaded. Ligplus software was used to analyze the two-dimensional interactions between the two proteins. PyMOL (version 4.3.0) software was used to visualize the amino acid residues involved in the interaction between the two proteins.
