# Supplementary material for: Reduction of eEF2 kinase alleviates the learning and memory impairment caused by acrylamide
Source: Cell Biosci. 2024 Aug 23;14:106. doi: 10.1186/s13578-024-01285-7 (PMC11344312; doi:10.1186/s13578-024-01285-7)
Supplement: Supplementary file 5 — Supplementary Material 5 [file 13578_2024_1285_MOESM5_ESM.docx]

**Supplementary Table 1. SD rat RT-qPCR primer sequences**

| **Name** | **Sequences** |
| --- | --- |
| **β-actin** | F: 5'-GACAGGATGCAGAAGGAGATTACT-3'  R: 5'-TGATCCACATCTGCTGGAAGGT-3' |
| **eEF2K** | F: 5'-AGGAGTATAATCGGCACAAGC-3'  R: 5'-GCGGACAAAGCCAGAGTT-3' |

**Supplementary Table 2. C57BL/6J mouse RT-qPCR primer sequences**

| **Name** | **Sequences** |
| --- | --- |
| **β-actin** | F: 5'-GGCTGTATTCCCCTCCATCG-3'  R: 5'-CCAGTTGGTAACAATGCCATGT-3' |
| **BDNF** | F：5'-TCATACTTCGGTTGCATGAAGG-3'  R: 5'-AGACCTCTCGAACCTGCCC-3' |
| **SYN1** | F：5'-CCAATCTGCCGAATGGGTACA-3'  R: 5'-GCGTTAGACAGCGACGAGAA-3' |
| **TrkB** | F：5'-CTGGGGCTTATGCCTGCTG-3'  R: 5'-AGGCTCAGTACACCAAATCCTA-3' |

**Supplementary Table 3. Downstream proteins regulated by eEF2K screened by mouse hippocampal proteomics.**

| **Gene name** | **KO +ACR vs WT +ACR Ratio** | **KO +ACR vs WT +ACR**  ***P* value** | **WT +ACR Regulated Type** | **KO +ACR Regulated Type** |
| --- | --- | --- | --- | --- |
| **Ace** | 2.009326807 | <0.001 | Down | Up |
| **Col18a1** | 1.348666976 | 0.015 | Down | Up |
| **Col1a2** | 1.834719027 | 0.03 | Down | Up |
| **Lpcat1** | 0.82030564 | <0.01 | Up | Down |
| **Gulp1** | 1.291623673 | <0.01 | Down | Up |
| **Acad8** | 1.305522085 | <0.001 | Down | Up |
| **Eif4ebp2** | 1.481735583 | 0.03 | Down | Up |
| **Cdc42ep2** | 0.69404085 | 0.02 | Down | Down |
