# Supplementary material for: Reduction of eEF2 kinase alleviates the learning and memory impairment caused by acrylamide
Source: Cell Biosci. 2024 Aug 23;14:106. doi: 10.1186/s13578-024-01285-7 (PMC11344312; doi:10.1186/s13578-024-01285-7)
Supplement: Supplementary file 6 — Supplementary Material 6 [file 13578_2024_1285_MOESM6_ESM.docx]

**Author statement**

WYH and WSY supervised the project, designed the experiments and analyzed the data; WXL performed the experiments, prepared the figures, and wrote part of the manuscript; WXL, ZRN, PYL and LZM contributed to the performance of the *in vivo* and *in vitro* experiments; WXL, ZRN, LZM, LYT, LHQ, ZFF and HXX contributed to the performance of the population study; PYL, YCP, MWW, YHW, WXY and LJJ wrote and edited the manuscript. All authors read and approved the final manuscript.
