# Supplementary figures and images for: Reduction of eEF2 kinase alleviates the learning and memory impairment caused by acrylamide
Source: Cell Biosci. 2024 Aug 23;14:106. doi: 10.1186/s13578-024-01285-7 (PMC11344312; doi:10.1186/s13578-024-01285-7)

Figure 5C


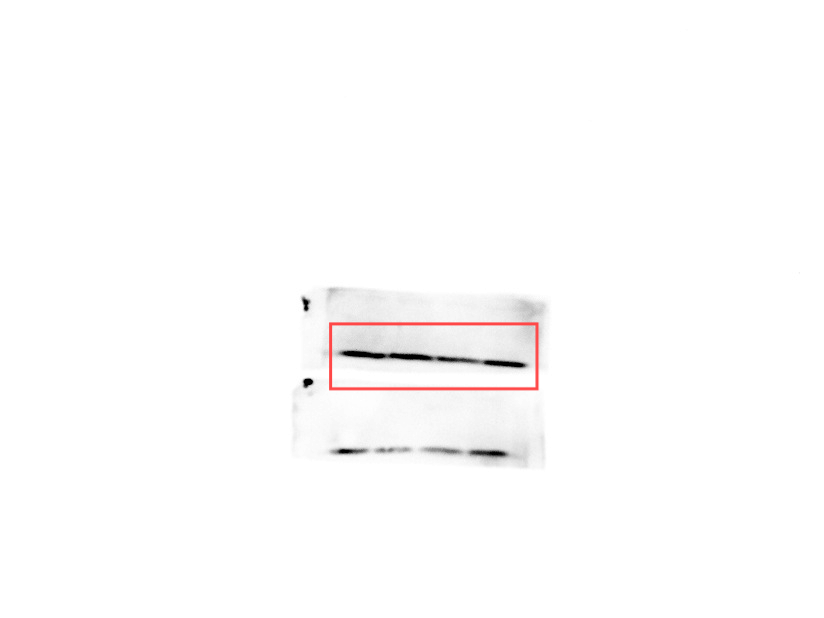


BDNF, 18 kDa


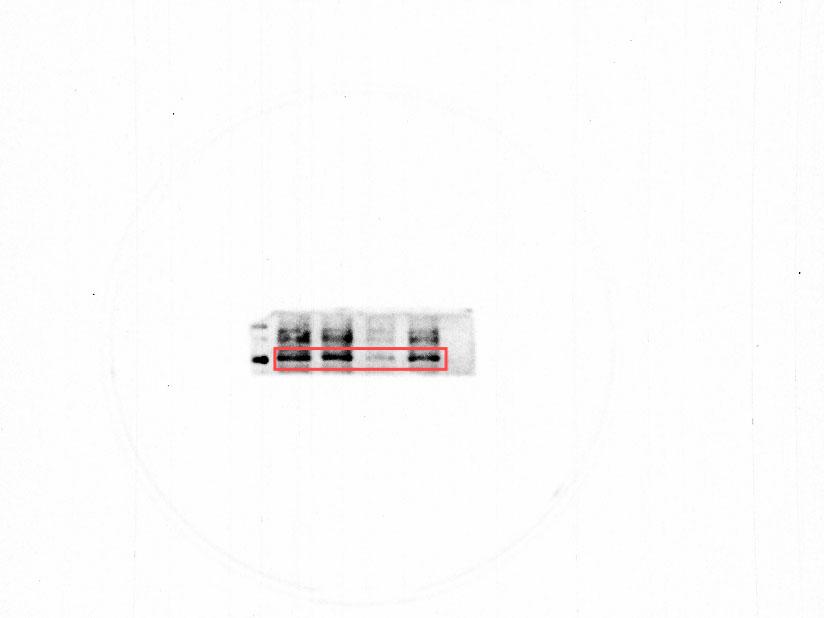


TrkB, 145 kDa


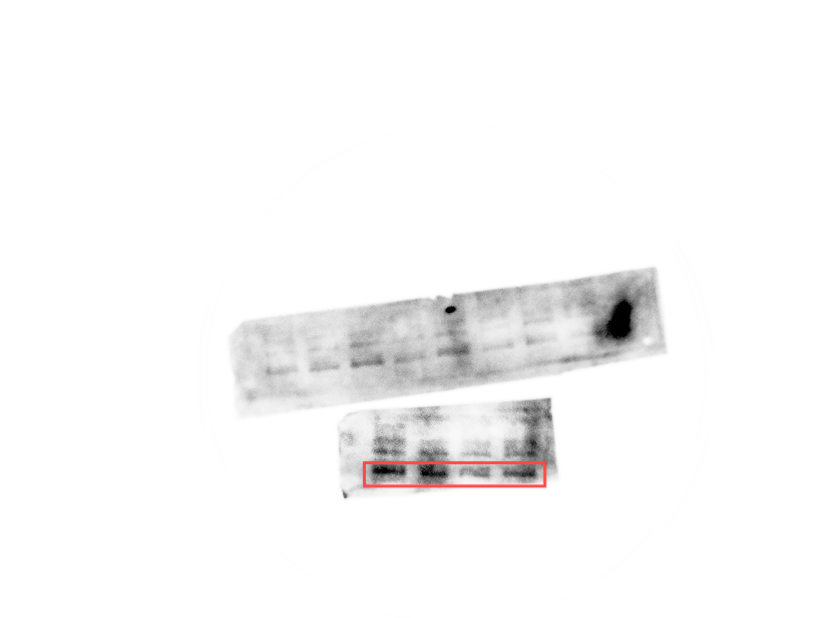


SYN1, 77 kDa


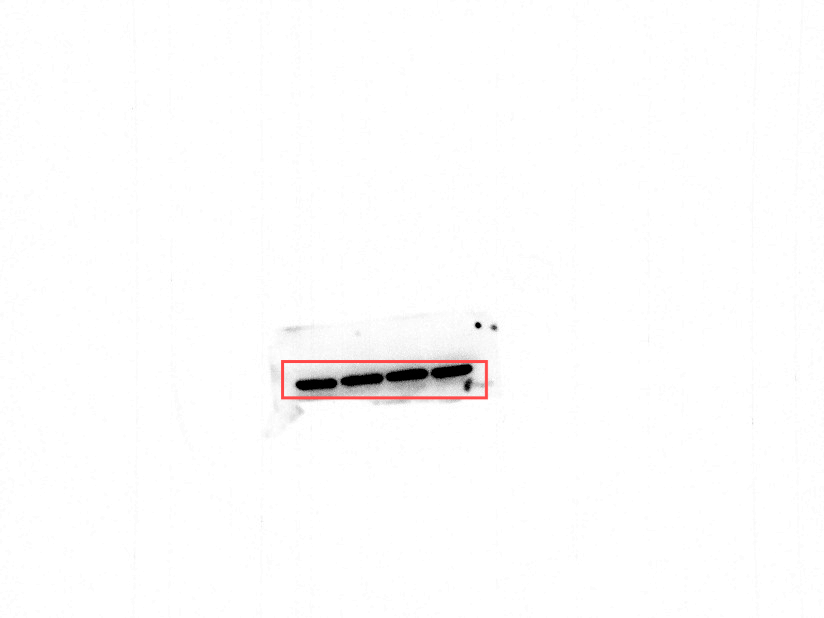


β-actin, 42 kDa

Supplement: Supplementary file 7 — Supplementary Material 7 [file 13578_2024_1285_MOESM7_ESM.docx]

Figure 6F


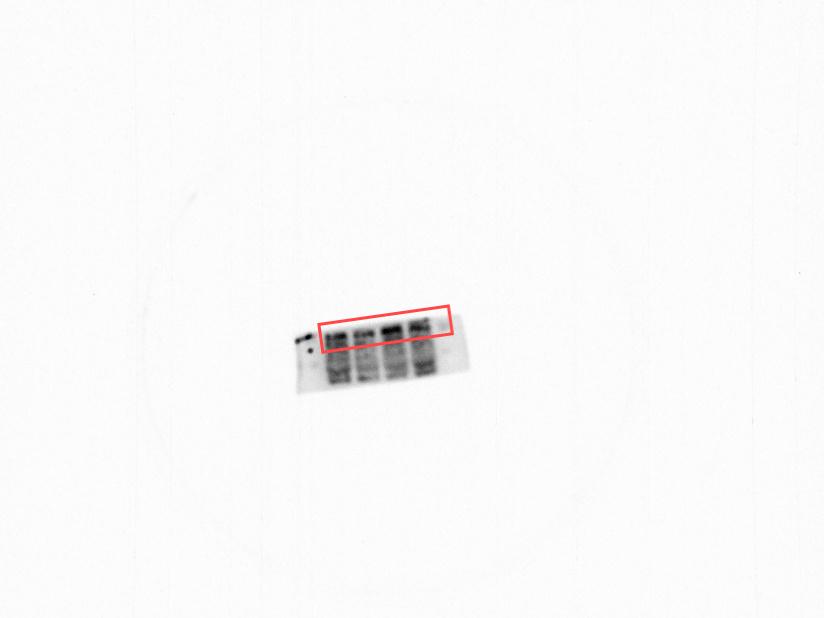


Lpcat1, 59 kDa

**
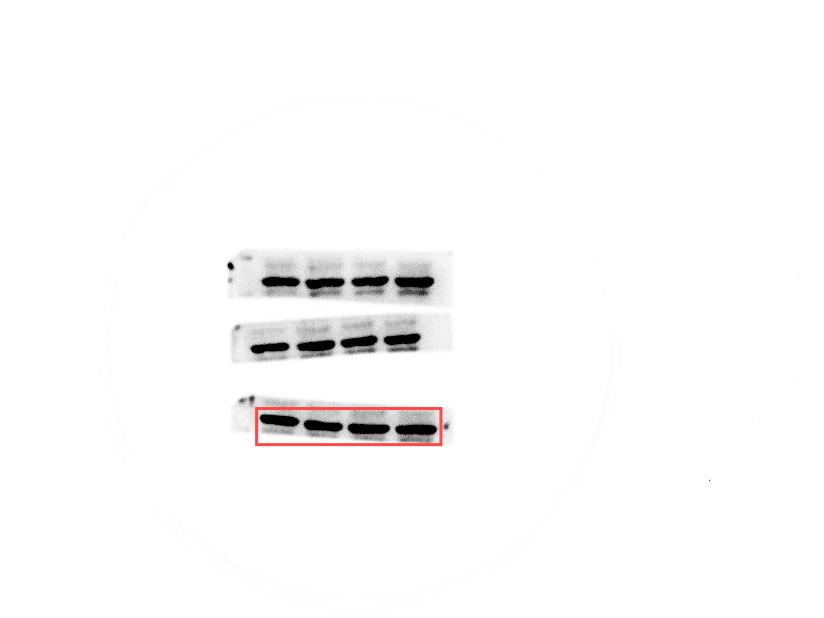
**

β-actin, 42 kDa

Supplement: Supplementary file 8 — Supplementary Material 8 [file 13578_2024_1285_MOESM8_ESM.docx]

Figure 1E


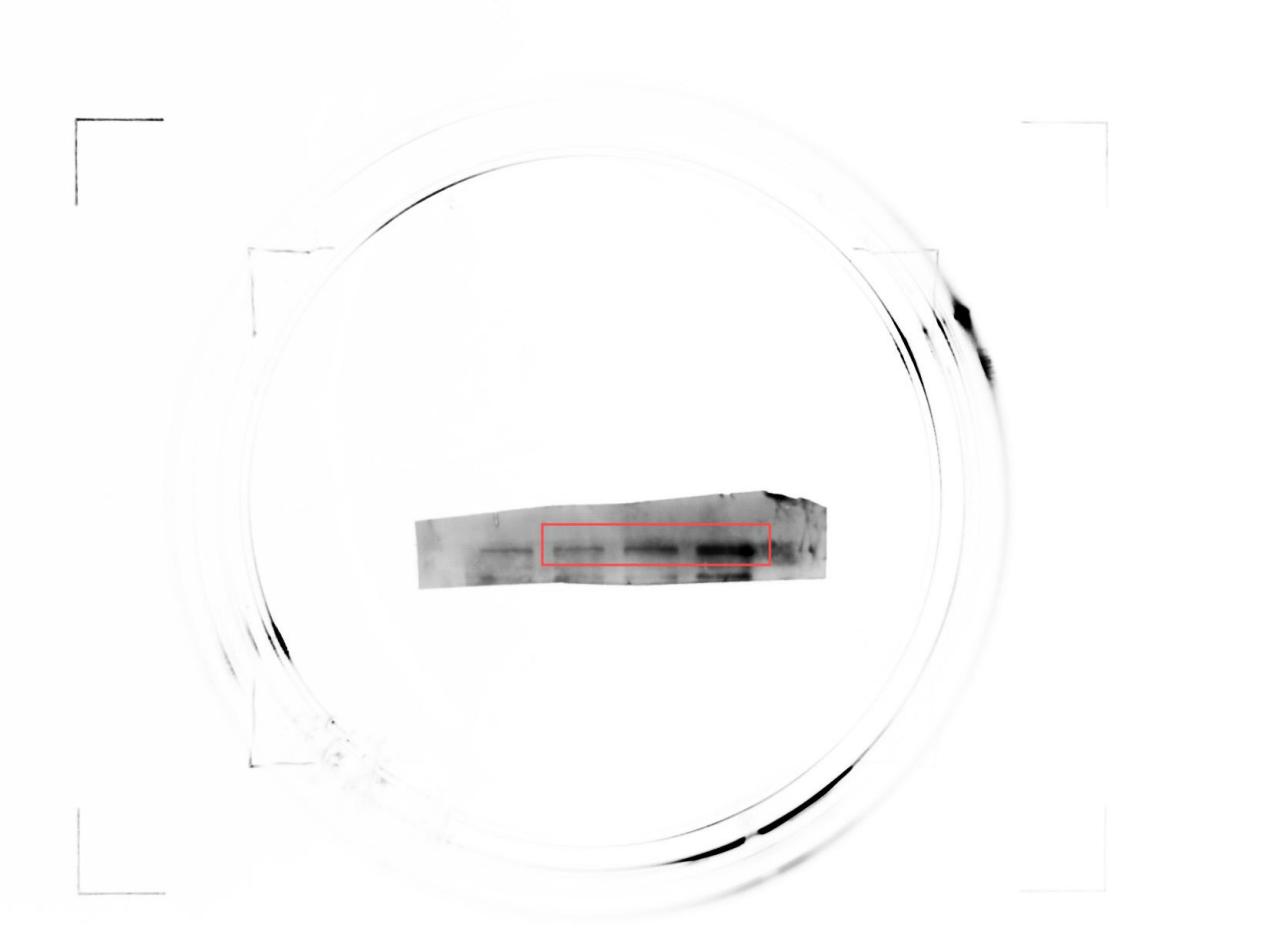


eEF2K, 105 kDa


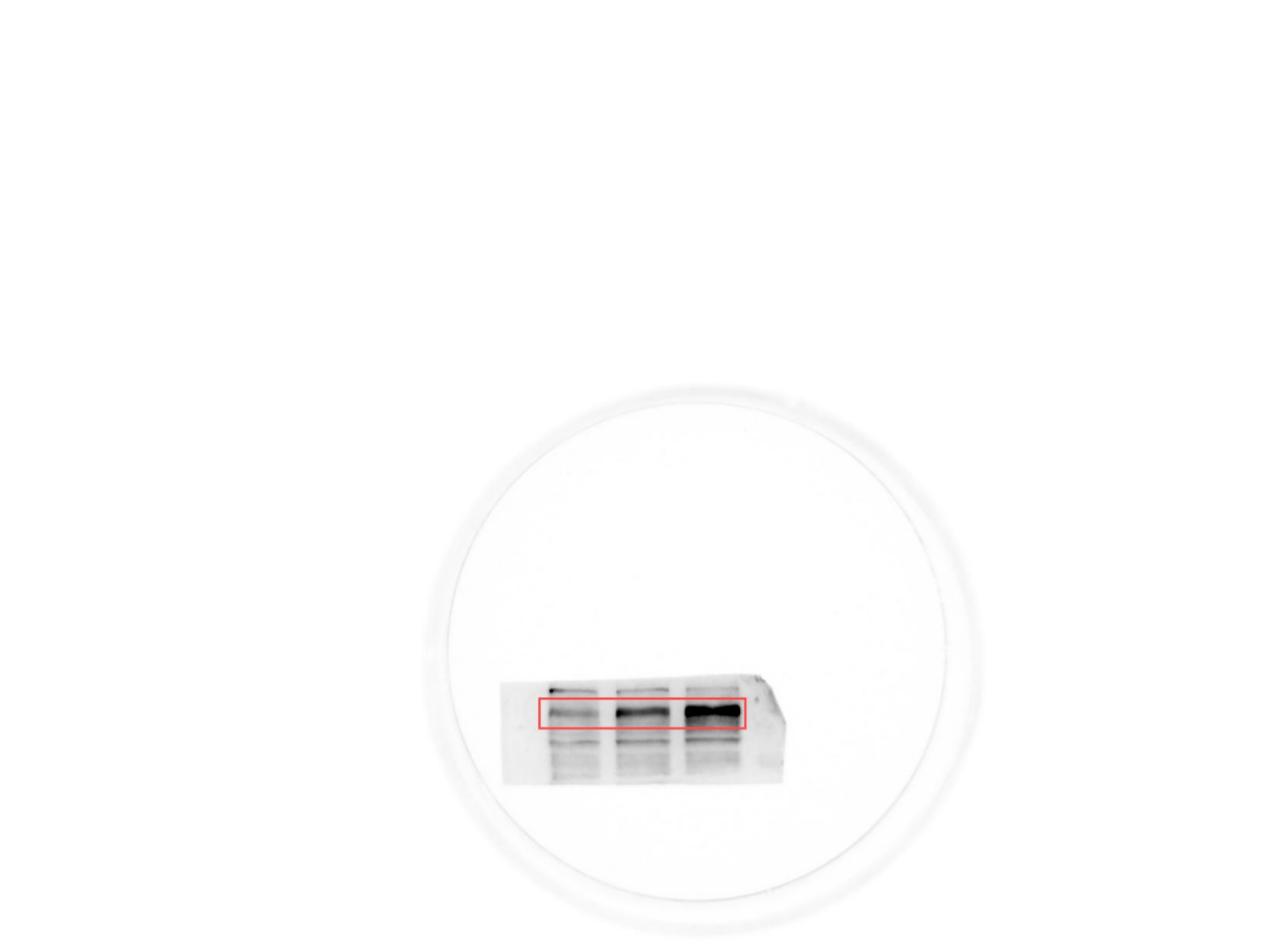


p- eEF2, 95 kDa


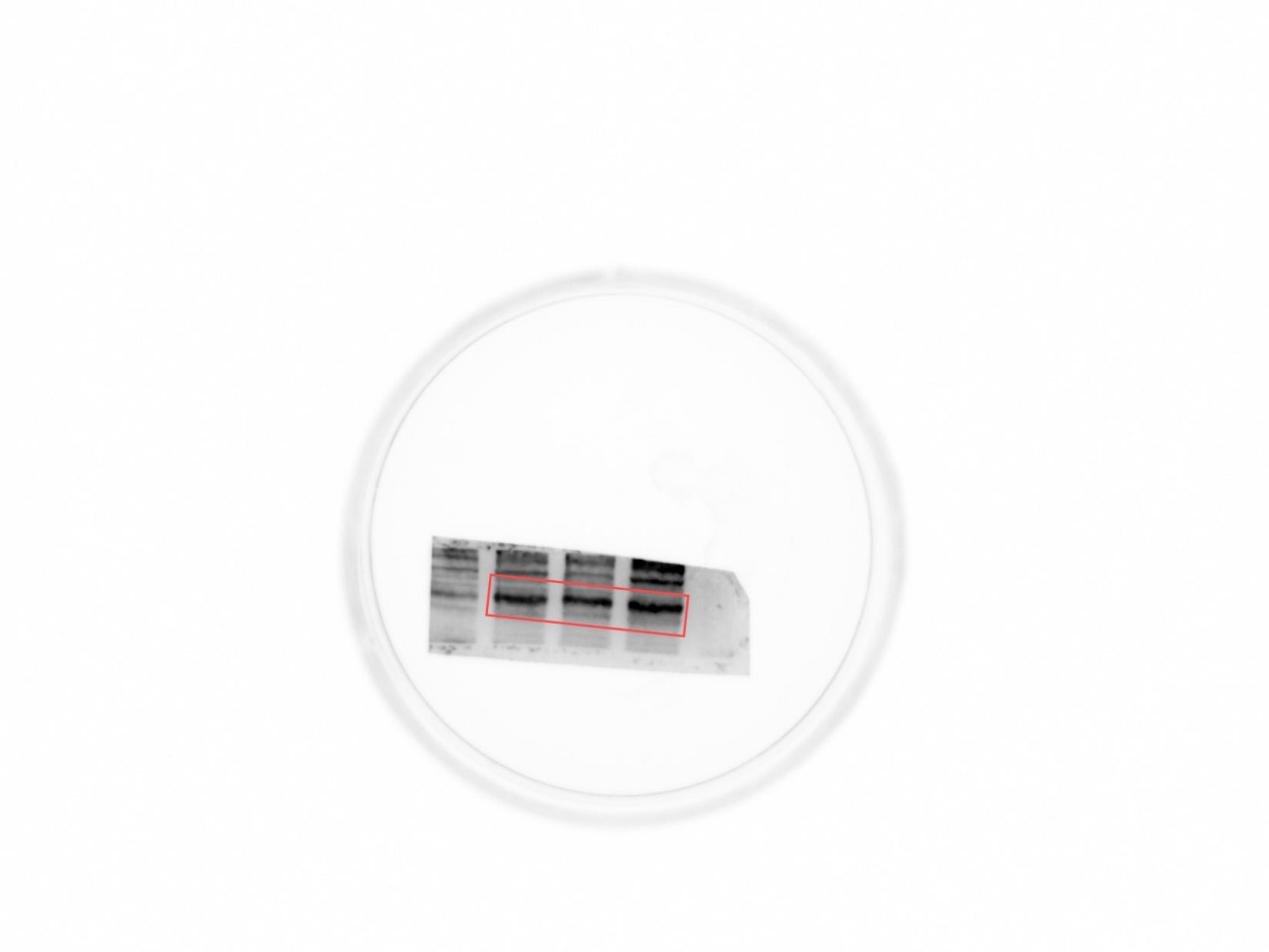


eEF2, 95kDa


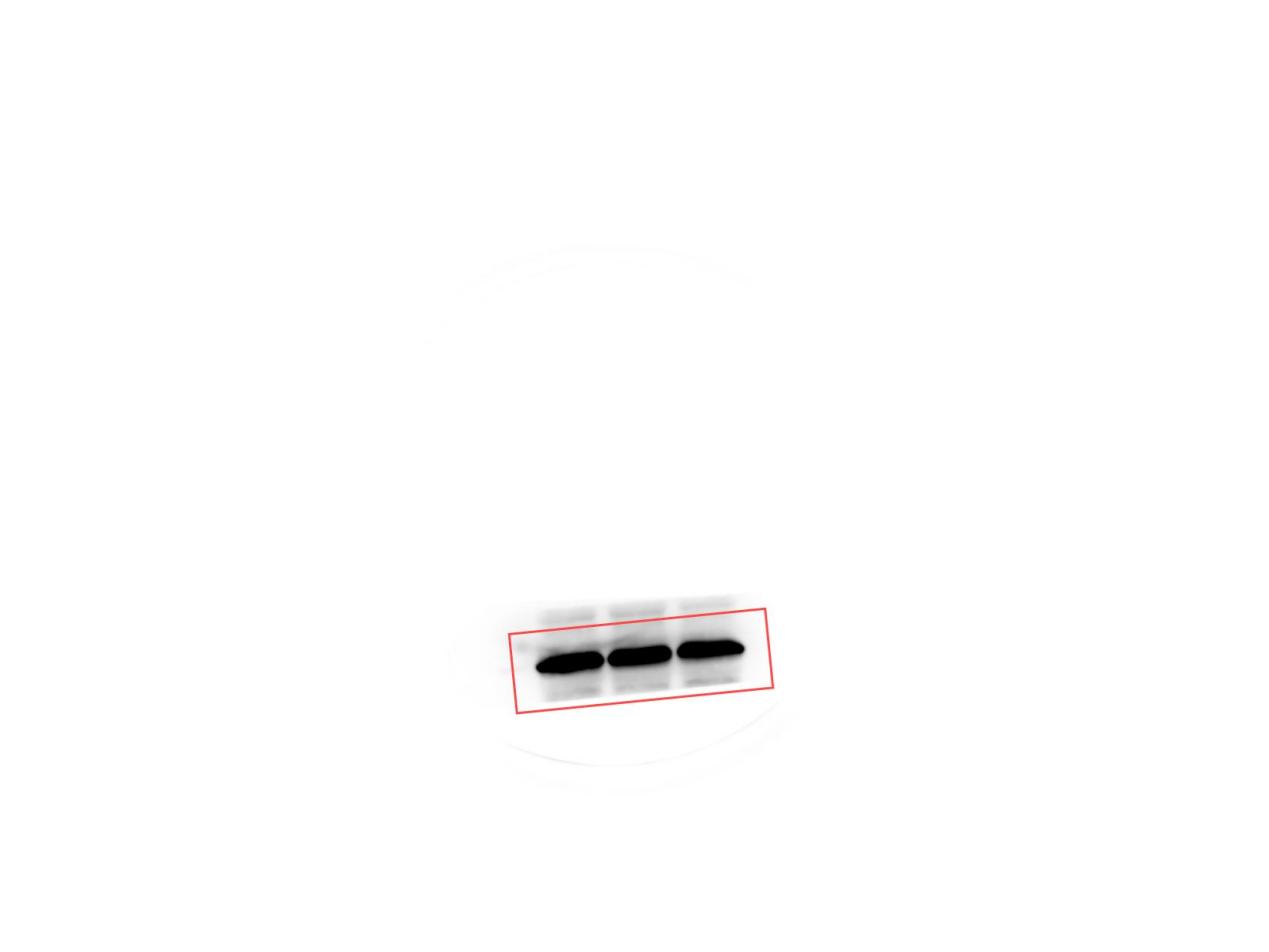


β-actin, 42 kDa

Supplement: Supplementary file 10 — Supplementary Material 10 [file 13578_2024_1285_MOESM10_ESM.docx]

Figure 2G


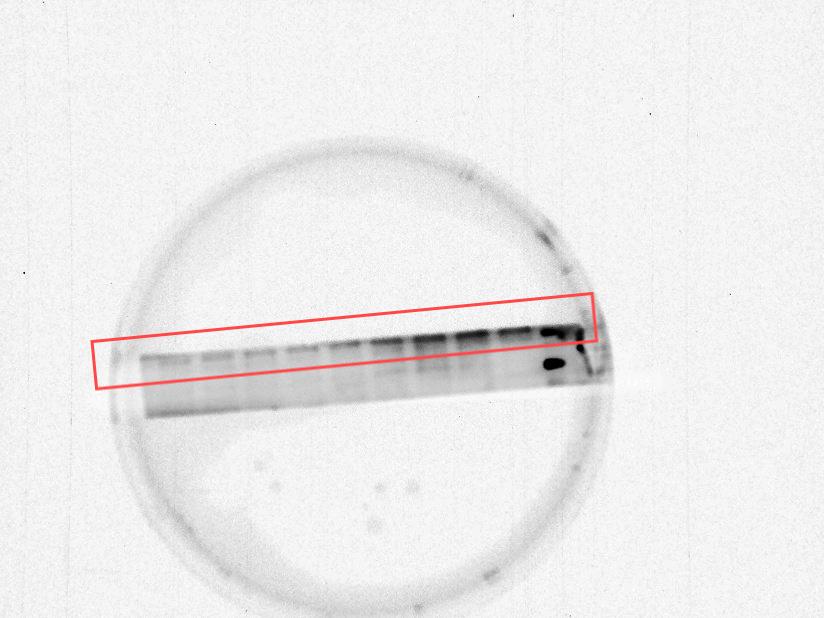


eEF2K, 105 kDa


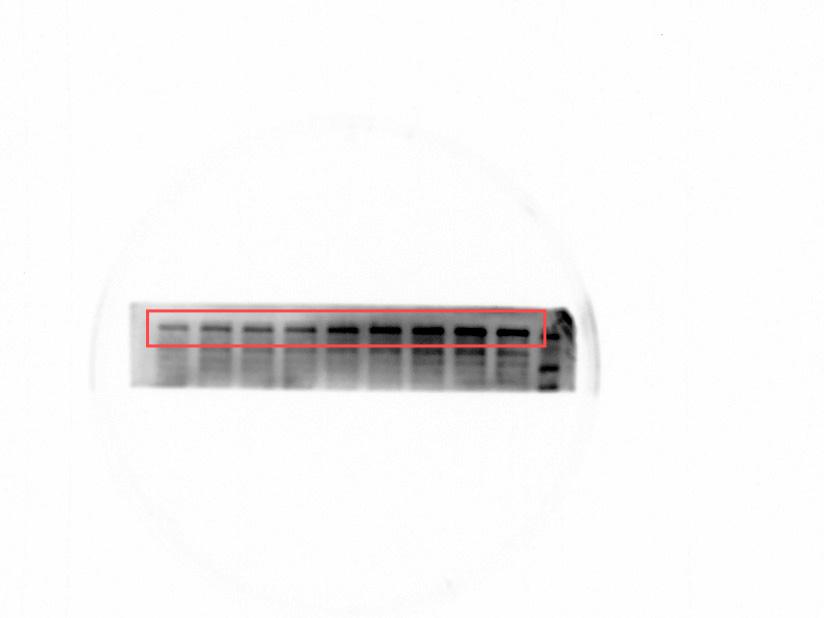


p- eEF2, 95 kDa


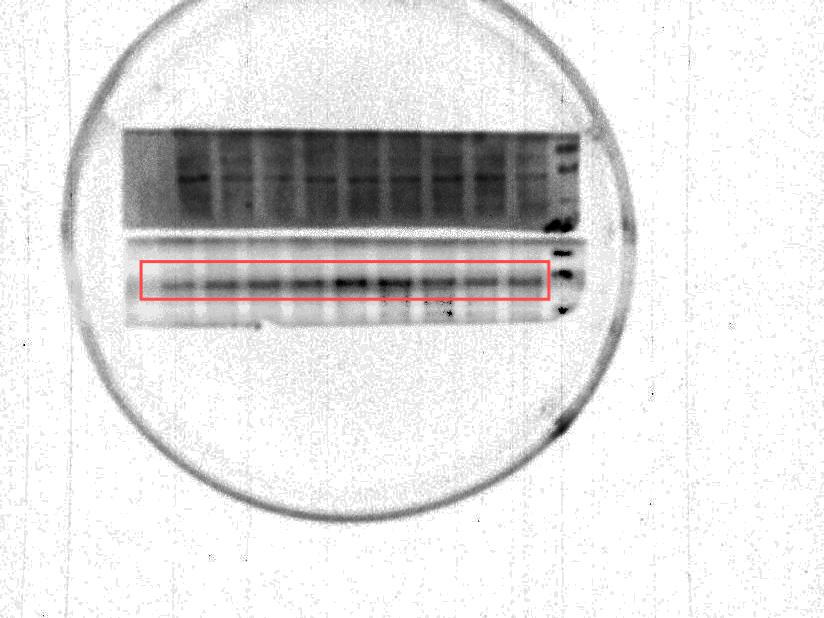


eEF2, 95 kDa


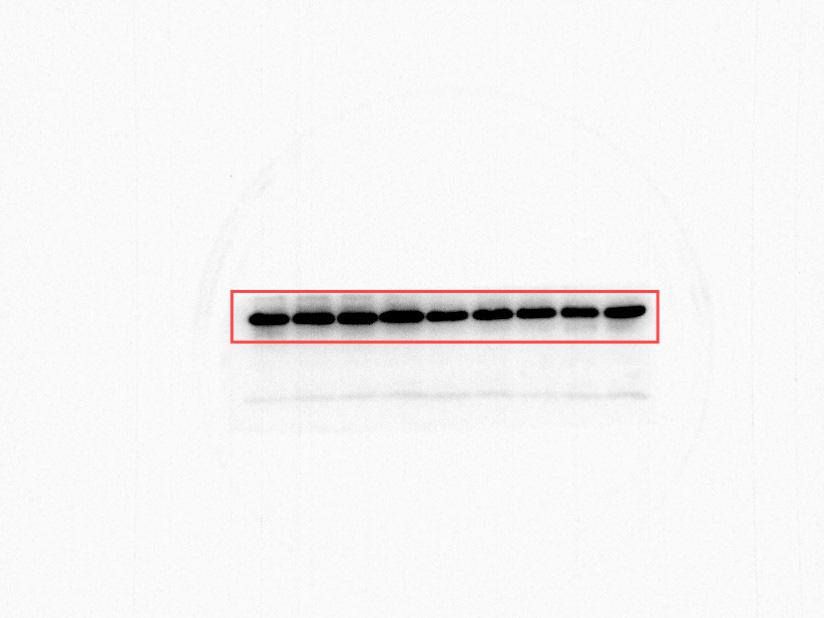


β-actin, 42 kDa

Supplement: Supplementary file 11 — Supplementary Material 11 [file 13578_2024_1285_MOESM11_ESM.docx]

Figure 3C


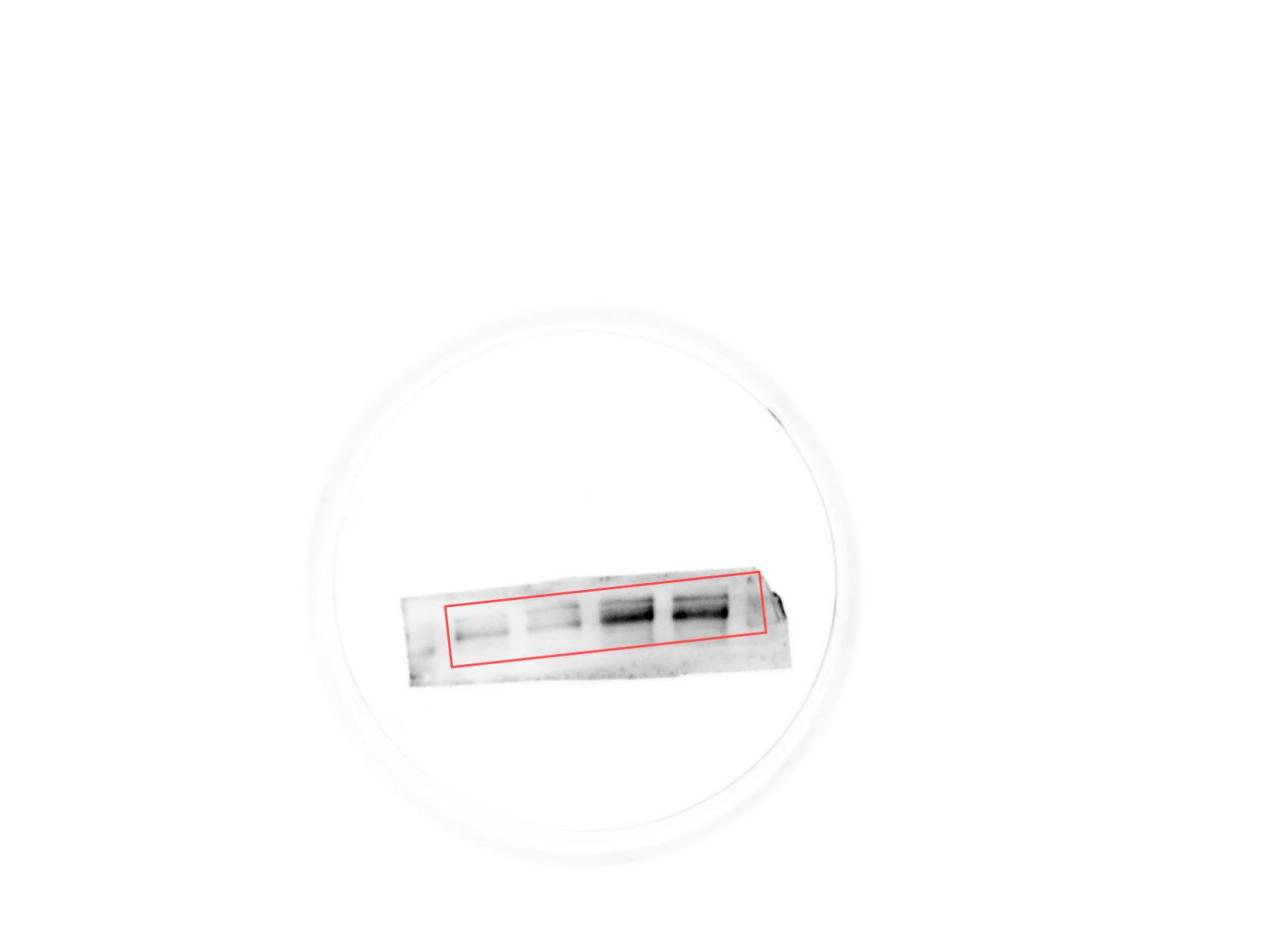


eEF2K, 105 kDa


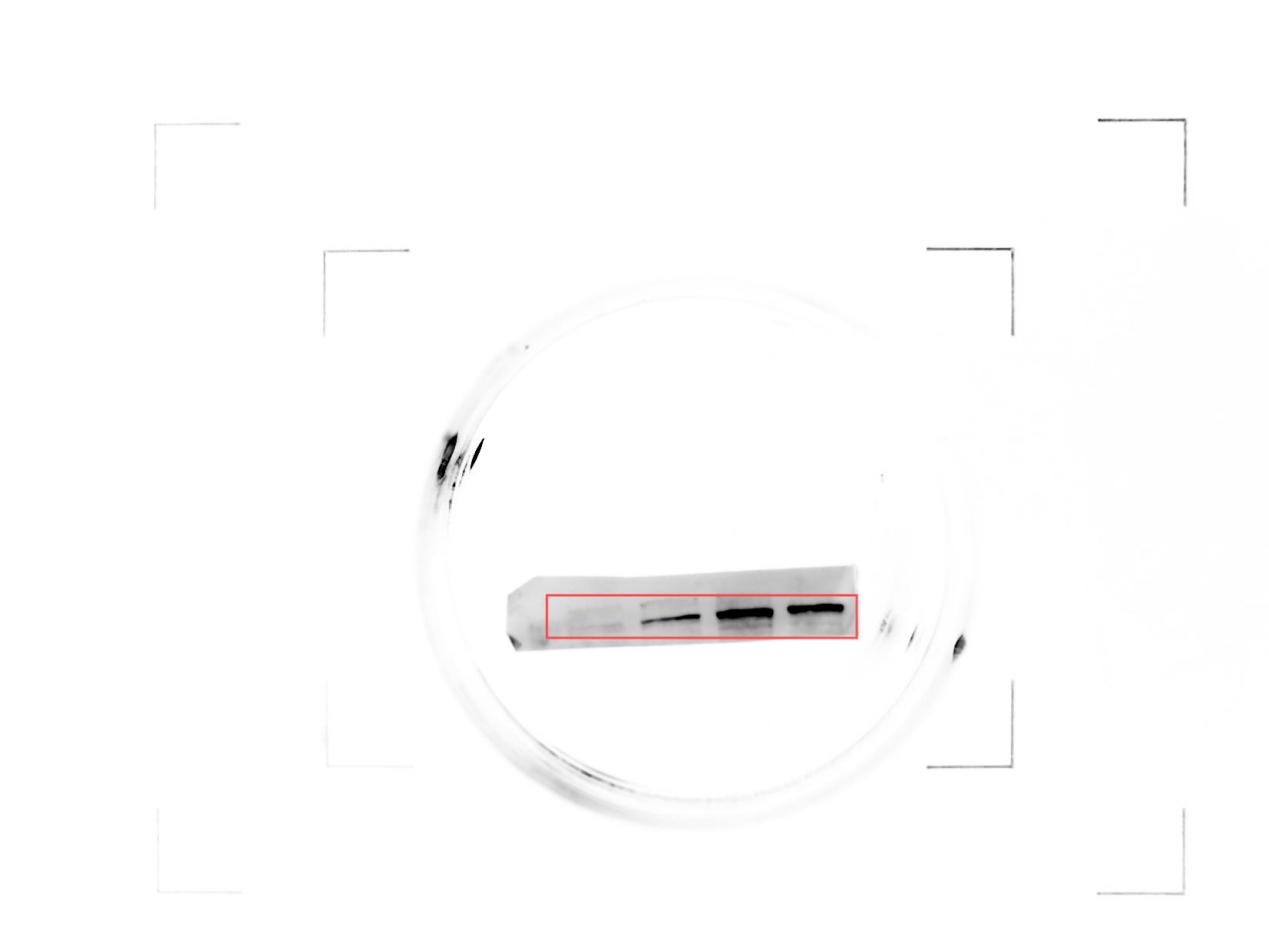


p- eEF2, 95 kDa


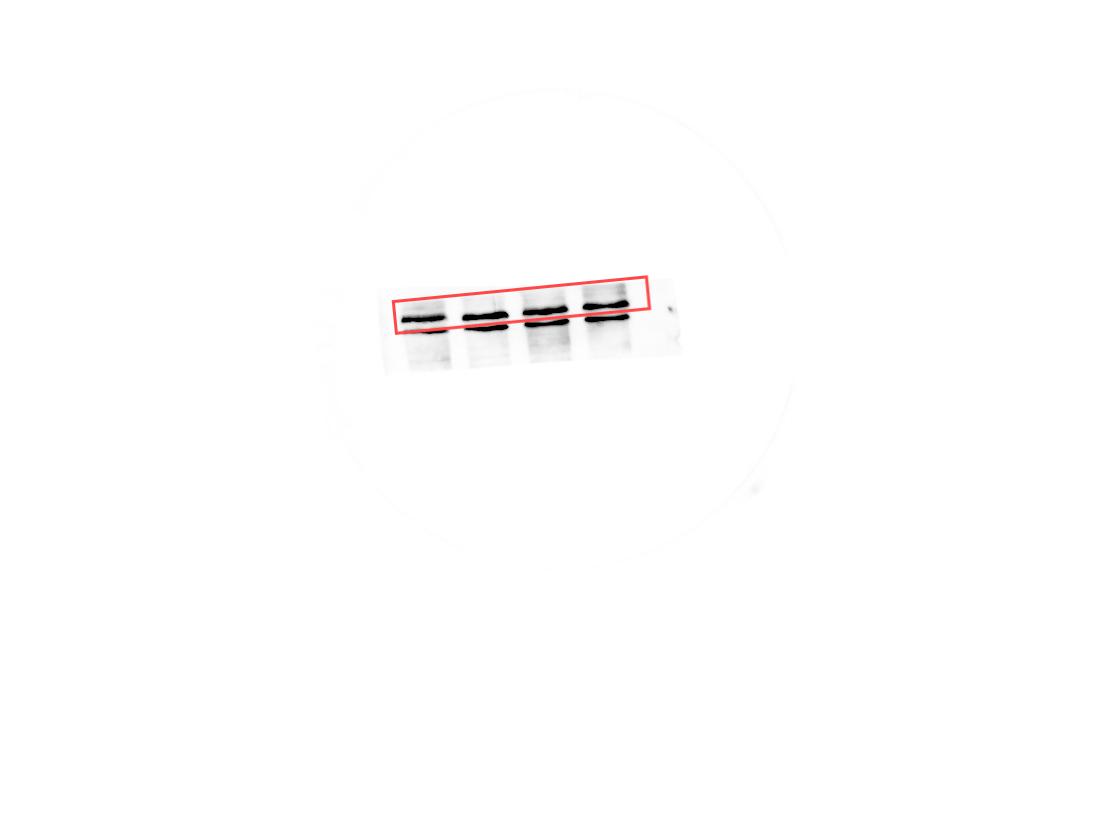


eEF2, 95 kDa


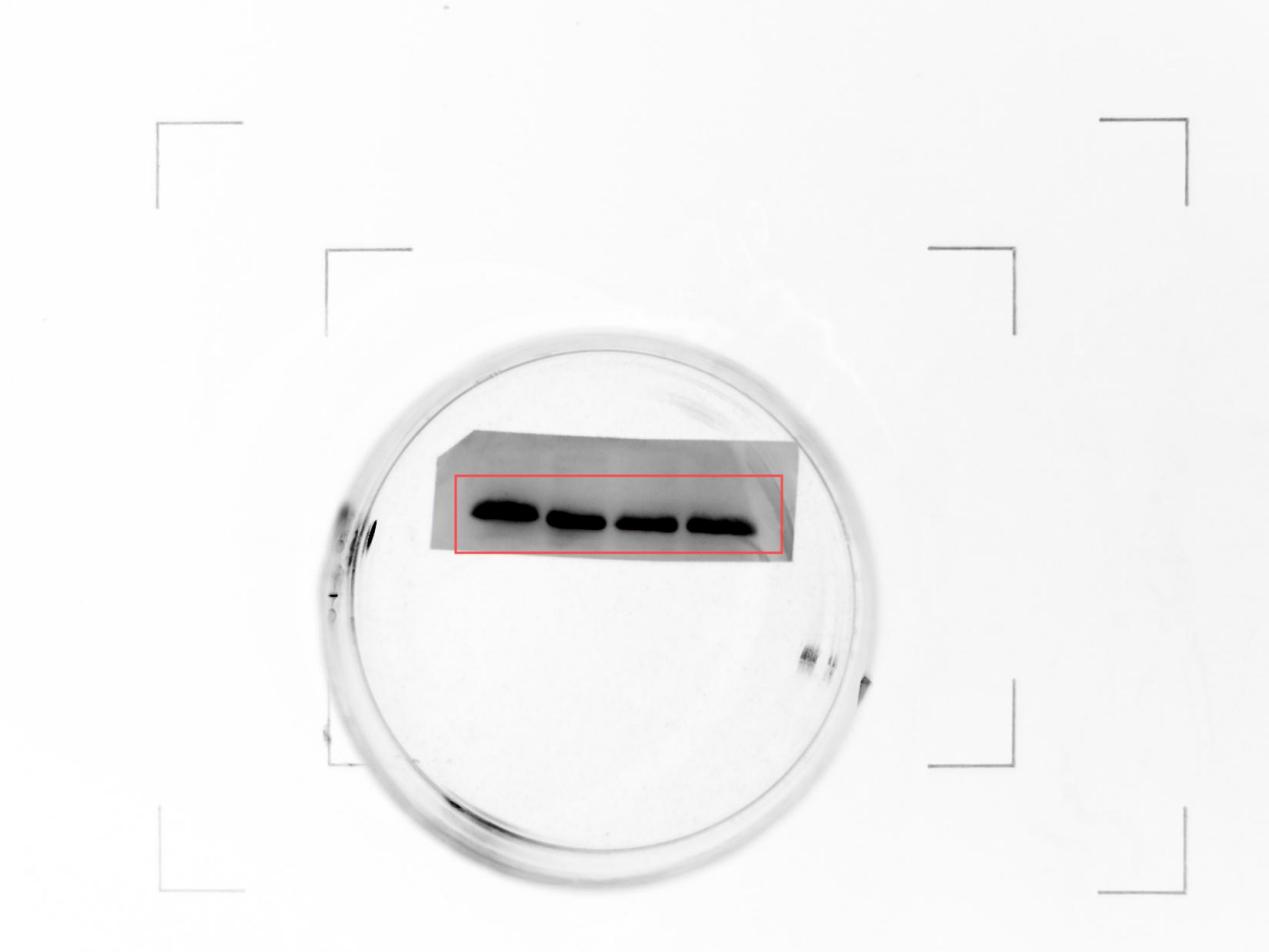


β-actin, 42 kDa

Supplement: Supplementary file 12 — Supplementary Material 12 [file 13578_2024_1285_MOESM12_ESM.docx]

Figure 3G


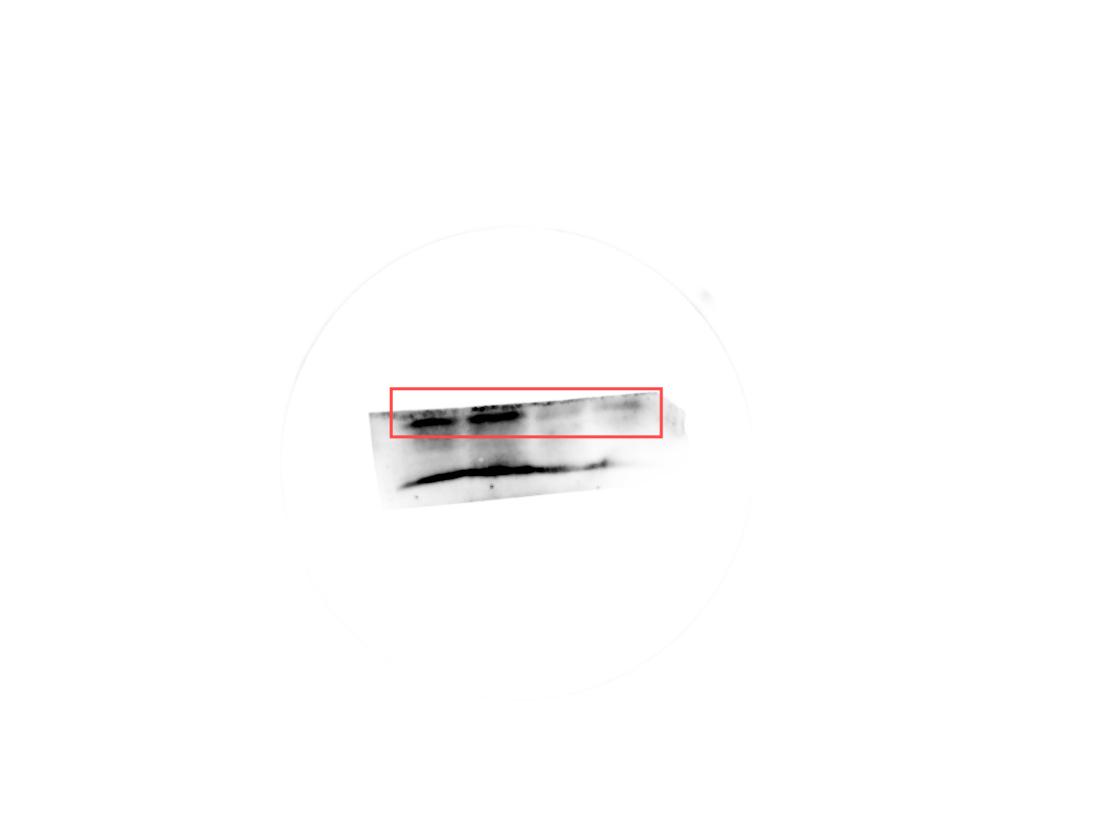


BDNF, 18 kDa


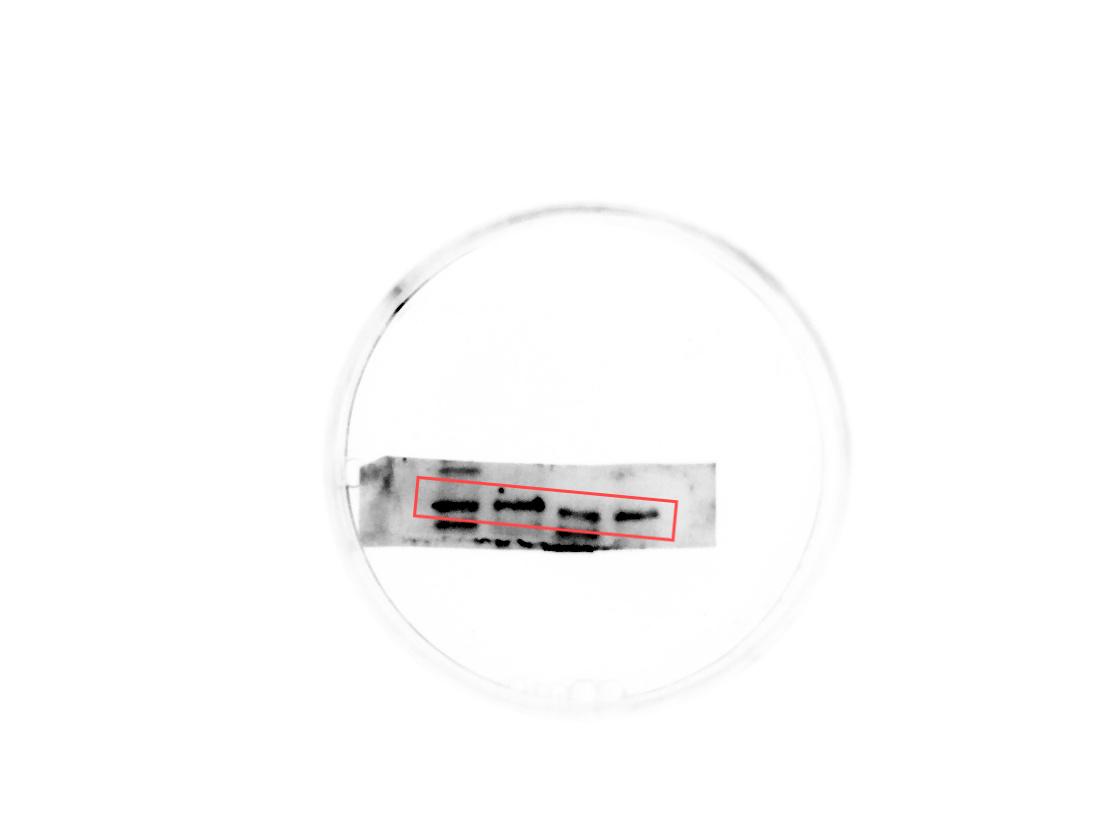


TrkB, 145 kDa


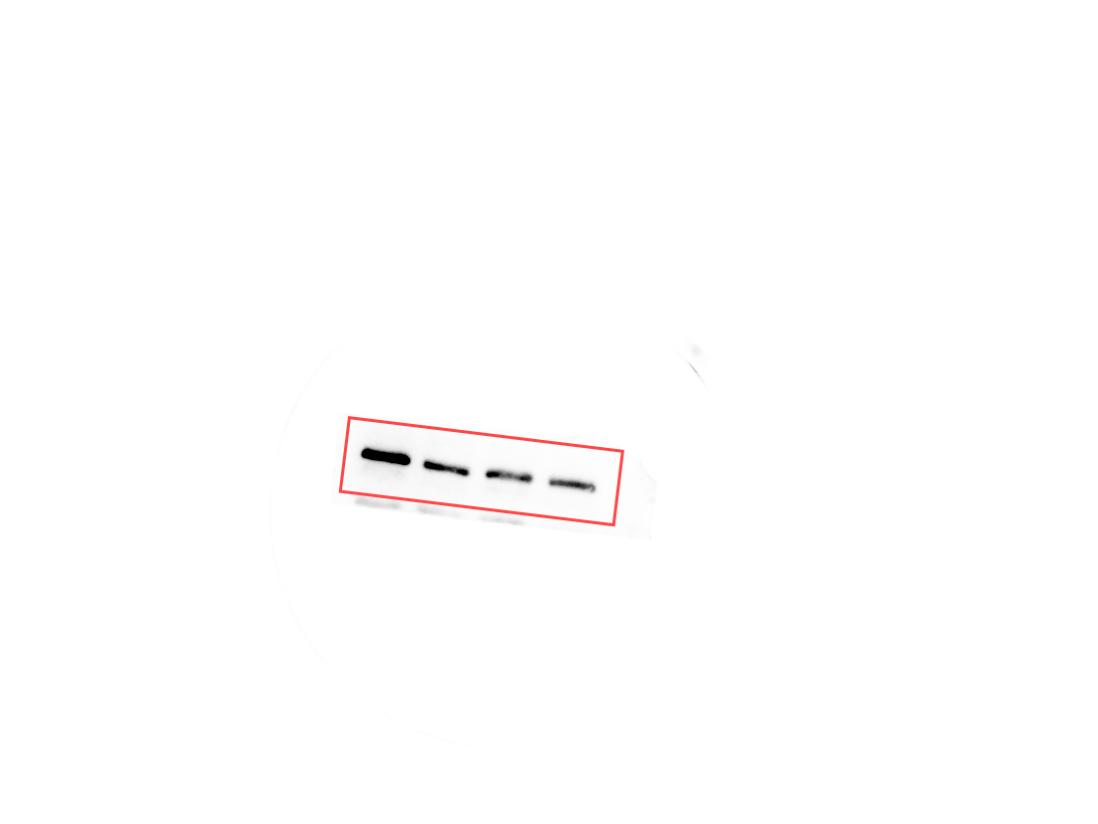


SYN1, 77 kDa


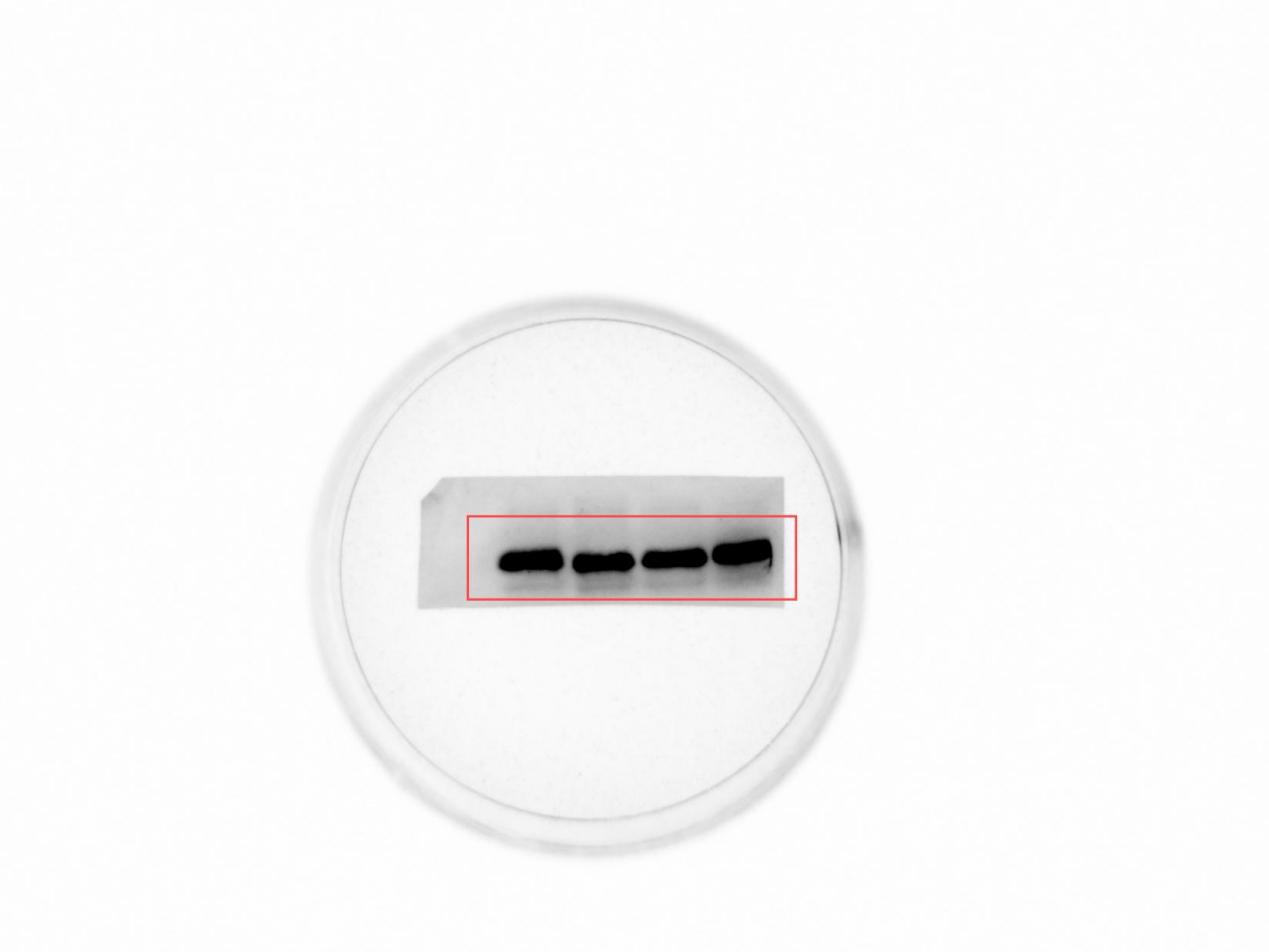


β-actin, 42 kDa

Supplement: Supplementary file 13 — Supplementary Material 13 [file 13578_2024_1285_MOESM13_ESM.docx]

Figure 4E


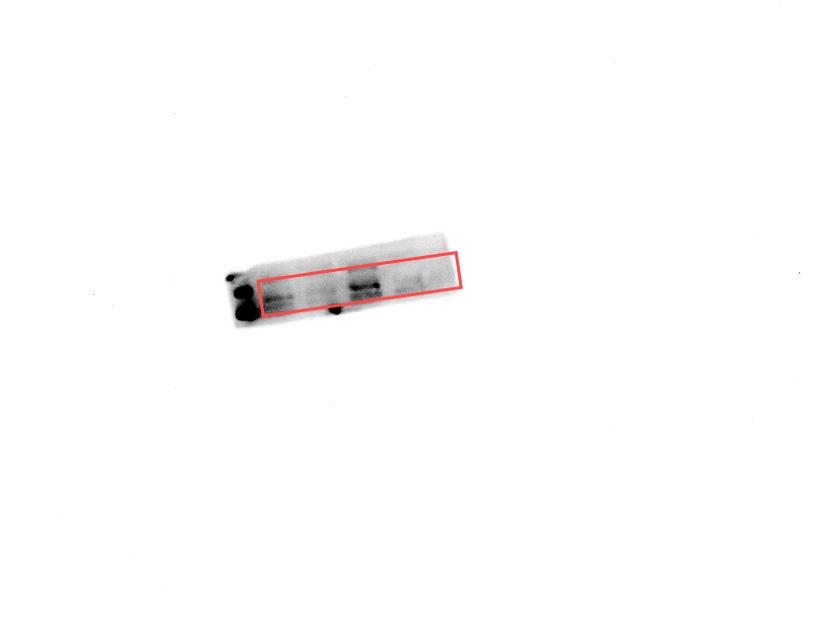


eEF2K, 105 kDa


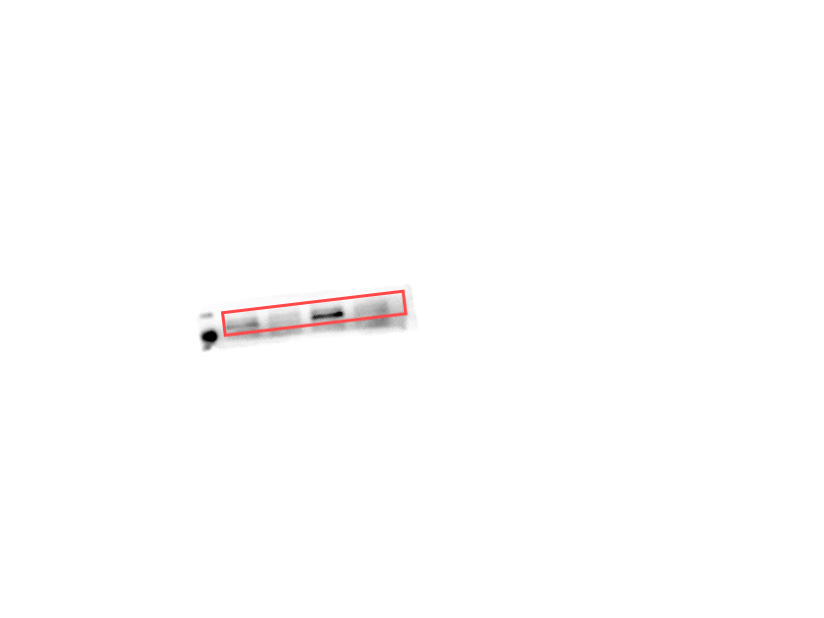


p- eEF2, 95 kDa


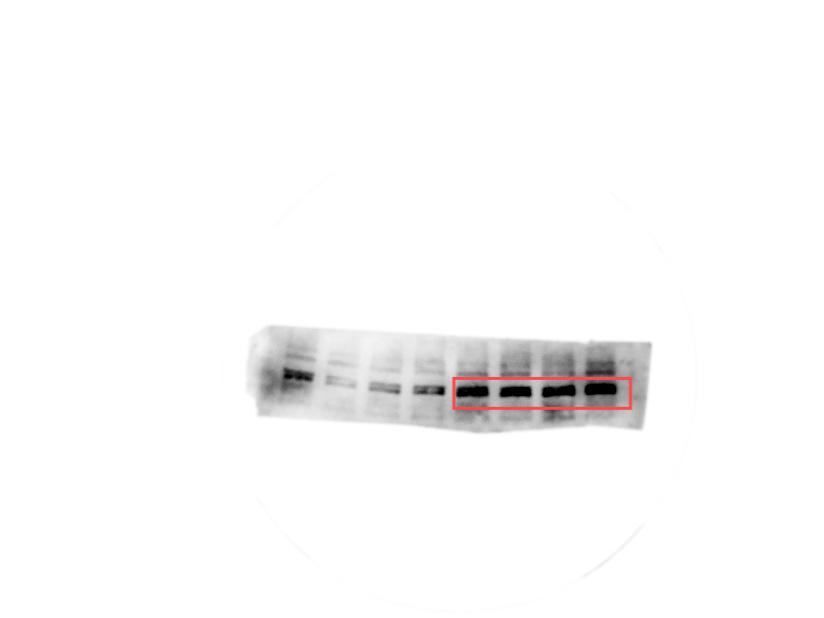


eEF2, 95 kDa


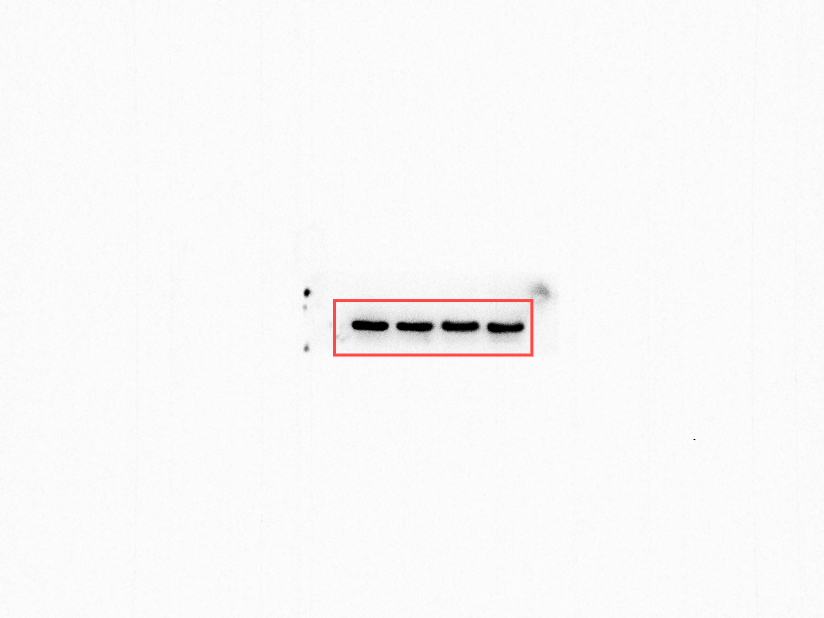


β-actin, 42 kDa

Supplement: Supplementary file 14 — Supplementary Material 14 [file 13578_2024_1285_MOESM14_ESM.docx]

**Graphical Abstract**


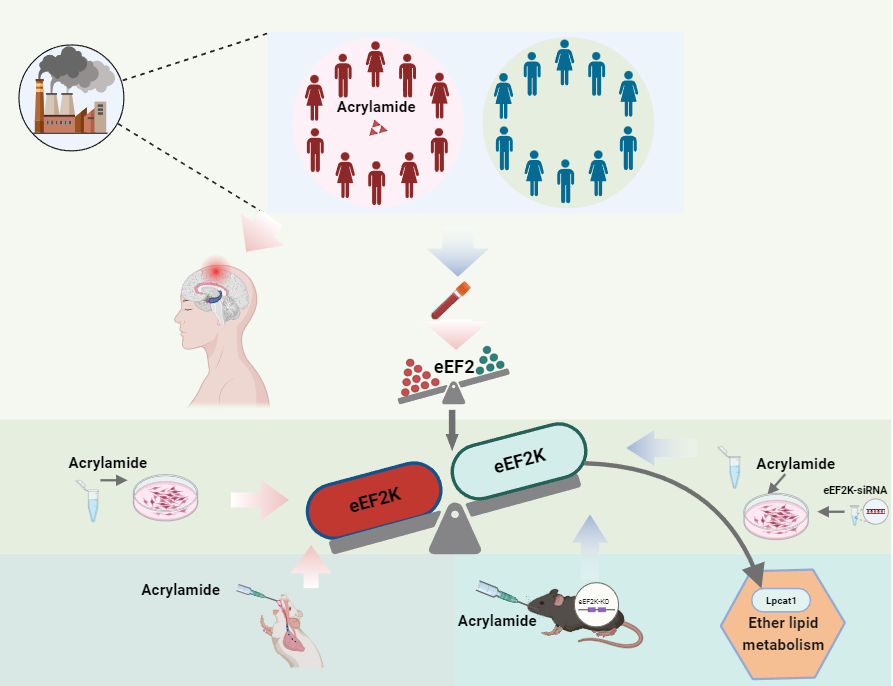

Supplement: Supplementary file 16 — Supplementary Material 16 [file 13578_2024_1285_MOESM16_ESM.docx]
