# Supplementary material for: Reduction of eEF2 kinase alleviates the learning and memory impairment caused by acrylamide
Source: Cell Biosci. 2024 Aug 23;14:106. doi: 10.1186/s13578-024-01285-7 (PMC11344312; doi:10.1186/s13578-024-01285-7)
Supplement: Supplementary file 15 — Supplementary Material 15 [file 13578_2024_1285_MOESM15_ESM.docx]

**Conflict of Interest**

The authors declare that they have no conflicts of interest relating to the content of this report.
