# Supplementary material for: Reduction of eEF2 kinase alleviates the learning and memory impairment caused by acrylamide
Source: Cell Biosci. 2024 Aug 23;14:106. doi: 10.1186/s13578-024-01285-7 (PMC11344312; doi:10.1186/s13578-024-01285-7)
Supplement: Supplementary file 19 — Supplementary Material 19 [file 13578_2024_1285_MOESM19_ESM.pdf]

49 Spadina Ave. Suite 200
Toronto ON M5V 2J1 Canada
www.biorender.com

Confirmation of Publication and Licensing Rights

August 7th, 2024
Science Suite Inc.

Subscription:	*Student Plan*
Agreement number:	*TN275IU3ME*
Journal name:	*Cell & Bioscience*

To whom this may concern,

This document is to confirm that Li Dongping has been granted a license to use the BioRender content, including icons, templates and other original artwork, appearing in the attached completed graphic pursuant to BioRender's Academic License Terms. This license permits BioRender content to be sublicensed for use in journal publications.

All rights and ownership of BioRender content are reserved by BioRender. All completed graphics must be accompanied by the following citation: “Created with BioRender.com”.

BioRender content included in the completed graphic is not licensed for any commercial uses beyond publication in a journal. For any commercial use of this figure, users may, if allowed, recreate it in BioRender under an Industry BioRender Plan.

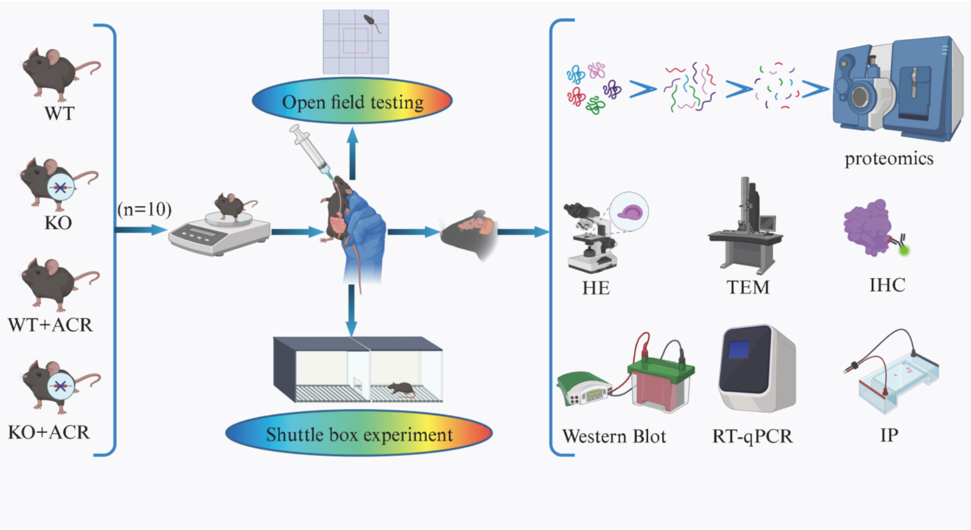

For any questions regarding this document, or other questions about publishing with BioRender refer to our BioRender Publication Guide, or contact BioRender Support at support@biorender.com.
